# Vaccine protection against rectal acquisition of SIVmac239 in rhesus macaques

**DOI:** 10.1371/journal.ppat.1008015

**Published:** 2019-09-30

**Authors:** Lucas Gonzalez-Nieto, Isabelle M. Castro, Georg F. Bischof, Young C. Shin, Michael J. Ricciardi, Varian K. Bailey, Christine M. Dang, Nuria Pedreño-Lopez, Diogo M. Magnani, Keisuke Ejima, David B. Allison, Hwi Min Gil, David T. Evans, Eva G. Rakasz, Jeffrey D. Lifson, Ronald C. Desrosiers, Mauricio A. Martins

**Affiliations:** 1 Department of Pathology, Miller School of Medicine, University of Miami, Miami, Florida, United States of America; 2 Department of Epidemiology and Biostatistics, Indiana University School of Public Health-Bloomington, Bloomington, Indiana, United States of America; 3 Wisconsin National Primate Research Center, University of Wisconsin, Madison, Wisconsin, United States of America; 4 Department of Pathology and Laboratory Medicine, University of Wisconsin-Madison, Madison, Wisconsin, United States of America; 5 AIDS and Cancer Virus Program, Frederick National Laboratory for Cancer Research, Frederick, Maryland, United States of America; Vaccine Research Center, UNITED STATES

## Abstract

A prophylactic vaccine against human immunodeficiency virus (HIV) remains a top priority in biomedical research. Given the failure of conventional immunization protocols to confer robust protection against HIV, new and unconventional approaches may be needed to generate protective anti-HIV immunity. Here we vaccinated rhesus macaques (RMs) with a recombinant (r)DNA prime (without any exogenous adjuvant), followed by a booster with rhesus monkey rhadinovirus (RRV)−a herpesvirus that establishes persistent infection in RMs (Group 1). Both the rDNA and rRRV vectors encoded a near-full-length simian immunodeficiency virus (SIVnfl) genome that assembles noninfectious SIV particles and expresses all nine SIV gene products. This rDNA/rRRV-SIVnfl vaccine regimen induced persistent anti-Env antibodies and CD8+ T-cell responses against the entire SIV proteome. Vaccine efficacy was assessed by repeated, marginal-dose, intrarectal challenges with SIVmac239. Encouragingly, vaccinees in Group 1 acquired SIVmac239 infection at a significantly delayed rate compared to unvaccinated controls (Group 3). In an attempt to improve upon this outcome, a separate group of rDNA/rRRV-SIVnfl-vaccinated RMs (Group 2) was treated with a cytotoxic T-lymphocyte antigen-4 (CTLA-4)-blocking monoclonal antibody during the vaccine phase and then challenged in parallel with Groups 1 and 3. Surprisingly, Group 2 was not significantly protected against SIVmac239 infection. In sum, SIVnfl vaccination can protect RMs against rigorous mucosal challenges with SIVmac239, a feat that until now had only been accomplished by live-attenuated strains of SIV. Further work is needed to identify the minimal requirements for this protection and whether SIVnfl vaccine efficacy can be improved by means other than anti-CTLA-4 adjuvant therapy.

## Author summary

Given the remarkable immune evasion properties of human immunodeficiency virus (HIV), unorthodox vaccine approaches might be needed to elicit protective anti-HIV immunity. Here we investigated whether vaccination with a near-full-length simian immunodeficiency virus (SIVnfl) genome could protect rhesus macaques (RMs) against pathogenic SIV challenge. To deliver SIVnfl, we used a DNA prime followed by a booster with a herpesvirus that establishes persistent infection in RMs. SIVnfl vaccinees developed long-lasting antibodies against the SIV Envelope protein and T-cell responses against the entire SIV proteome. Encouragingly, SIVnfl vaccinees were significantly protected against intrarectal challenge with SIVmac239, a feat that until now had only been accomplished by live-attenuated strains of SIV. Curiously, combining the aforementioned vaccine regimen with immune checkpoint blockade did not result in protection against SIVmac239 infection in a separate group of RMs. Vaccination with inactivated immunodeficiency virus genomes should, therefore, be considered as a potential strategy for eliciting anti-HIV immunity.

## Introduction

Human immunodeficiency virus (HIV) continues to infect thousands of new people every day, despite advances in prevention modalities and antiretroviral therapy coverage [1]. Mathematical models have suggested that combining current HIV prevention and treatment strategies with a prophylactic HIV vaccine could significantly restrict the growth of the HIV pandemic [2]. Unfortunately, however, developing such a vaccine has been exceedingly difficult, as seen by the failure of most HIV vaccine trials conducted to date [3–7]. Although the RV144 trial remains the only report of vaccine-mediated reduction in HIV infection rates [8], the observed results were modest, short-lived, and continue to be questioned [9, 10]. Given the refractoriness of HIV to immune responses induced by conventional immunization protocols, new or unorthodox approaches may be needed to generate protective anti-HIV immunity.

One non-conventional strategy that holds great promise is the use of live herpesviruses to deliver HIV antigens. Because herpesviruses establish persistent infections that remain largely subclinical in their hosts, a herpesvirus-based HIV vaccine could promote chronic low-level exposure to HIV antigens, a feature that might facilitate the induction of long-term protective anti-HIV immunity. We have recently generated recombinant (r) forms of the gamma-herpesvirus rhesus monkey rhadinovirus (RRV) containing a near-full-length simian immunodeficiency virus (SIVnfl) genome [11]. SIVnfl expresses all nine SIV gene products and assembles noninfectious SIV particles. Importantly, rhesus macaques (RMs) inoculated with a rRRV-SIVnfl vector became persistently infected, mounted durable anti-Env antibody (Ab) responses, and developed effector-differentiated SIV-specific CD8+ T-cells [11]. Of note, rRRV-SIVnfl vaccination induced CD8+ T-cell responses against all nine SIV proteins, consistent with the ability of the SIVnfl insert to express the entire SIVnfl proteome [11]. Crucially, these features are also observed following inoculation with live-attenuated SIV strains, the most effective vaccine modality against pathogenic SIV challenge in nonhuman primates.

Despite these encouraging results, the magnitude of SIV-specific immune responses induced by rRRV-SIVnfl vaccination was low compared to what is seen after live-attenuated SIV inoculation. This prompted us to test whether booster immunizations with SIVnfl-expressing rDNA plasmids [delivered by intramuscular (IM) electroporation (EP)] could amplify SIV-specific immune responses in rRRV-SIVnfl-primed RMs. We evaluated this possibility in a recent study, where rRRV-SIVnfl-primed RMs received a series of four rDNA-SIVnfl boosters given three weeks apart. The first two rDNA-SIVnfl boosters were administered without any exogenous adjuvants. However, in an attempt to augment vaccine immunogenicity, the third and fourth rDNA-SIVnfl boosters were followed by an infusion of the monoclonal (m)Ab Ipilimumab (Ipi), which blocks the immune checkpoint receptor cytotoxic T-lymphocyte antigen-4 (CTLA-4) [12]. CTLA-4 is upregulated on T-cells shortly after activation and suppresses immune responses by interfering with CD28-mediated signaling, a key step for T-cell activation [13]. Consequently, blocking CTLA-4 *in vivo* can enhance adaptive immune responses. Indeed, the ability of anti-CTLA-4 therapy to enhance anti-tumor immunosurveillance has led to the approval of Ipi as a drug against advanced melanoma [12, 14]. Of note, previous studies have shown that anti-CTLA-4 can also amplify adaptive immune responses induced by prophylactic vaccination [15–17]. The aforementioned rDNA-SIVnfl booster vaccinations significantly expanded SIV-specific T-cell responses and Env-specific Ab responses in the rRRV-SIVnfl-primed RMs. Importantly, this rRRV/rDNA-SIVnfl vaccine regimen afforded significant protection against repeated, marginal-dose, intravenous (IV) challenges with SIVmac239 [18]. However, because that study lacked an Ipi-untreated vaccine arm, we could not delineate the contribution of the Ipi infusions to the outcome of the rRRV/rDNA-SIVnfl vaccine trial.

Here we sought to expand upon our recent findings by assessing if SIVnfl vaccination can protect RMs against rectal challenges with SIVmac239. Because DNA vaccines are typically used to prime immune responses in mixed-modality immunization protocols, we used the same SIVnfl-expressing vectors described above in a rDNA-prime/rRRV-boost configuration. Given that our previous study left some unanswered questions regarding the role of CTLA-4 blockade on vaccine performance, we characterized the immunogenicity and efficacy of the rDNA/rRRV-SIVnfl vaccine regimen in the absence (Group 1) or presence (Group 2) of anti-CTLA-4 therapy during the priming phase.

## Results

### Experimental design

The sixteen RMs in Groups 1 and 2 were each primed with two rDNA-SIVnfl plasmids (administered by IM EP) that differed only in the Env protein expressed by each plasmid (S1A and S1B Fig). The SIVnfl insert in vector 1 expressed a truncated version of SIVmac239 Env (E_767_Stop) intended to increase Env incorporation into the non-infectious SIVnfl virions (S1A Fig) [19], while the SIVnfl insert present in vector 2 expressed an intact SIVmac316 Env protein (S1B Fig). SIVmac316 is a neutralization-sensitive derivative of SIVmac239 [20]. The Env proteins of these two SIV clones differ from each other in only eight amino acids [20]. Given the poor immunogenicity of SIVmac239 Env (closed conformation), the use of SIVmac316 Env (open conformation) was intended to expose potential neutralizing epitopes that would normally be occluded in the SIVmac239 Env spike. Furthermore, both vectors 1 and 2 contained a 6-base pair (bp) deletion in *nef* (S1A and S1B Fig), corresponding to amino acids 239–240, that abrogates Nef-mediated major histocompatibility complex class-I (MHC-I) down-regulation [21]. Additionally, the *tat* gene in vectors 1 and 2 encoded a L_35_Q substitution within the Mamu-A*01-restricted Tat_28-35_SL8 epitope that severely reduces its affinity for the Mamu-A*01 molecule (S1A and S1B Fig) [22]. Following SIVmac239 infection, Mamu-A*01+ RMs tend to mount a vigorous Tat_28-35_SL8-specific CD8+ T-cell response that is rapidly rendered ineffective by the selection of SIV “escape” variants [22]. This selection occurs with no apparent fitness cost to the virus [22, 23]. Because subdominant CD8+ T cell responses can be actively suppressed by dominant CD8+ T cell responses in the context of DNA immunization [24], the rationale for the Tat L_35_Q change in the rDNA-SIVnfl vectors was to prevent the priming of Tat_28-35_SL8-specific CD8+ T-cells. Our hope was that this strategy would broaden the repertoire of vaccine-induced SIV-specific CD8+ T cells in the Mamu-A*01+ RMs used in the present study.

**Fig 1:**
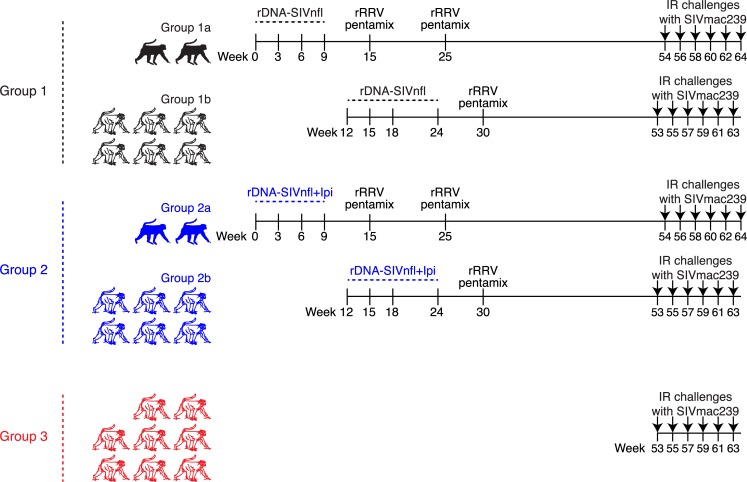
Experimental layout. The 16 RMs in Group 1 (1a+1b) and Group 2 (2a+2b) were primed four times with SIVnfl-expressing rDNA-SIVnfl plasmids delivered by IM electroporation. The vaccinees in Groups 2a and 2b, but not those in Groups 1a and 1b, received infusions of Ipi (3.0 mg/kg/dose) on the day after each rDNA-SIVnfl prime. All vaccinated animals in Group 1 (1a+1b) and Group 2 (2a+2b) were boosted with the same mixture of five rRRV vectors (i.e., rRRV pentamix) six weeks after the 4^th^ DNA prime. Details on the inserts delivered by each rDNA and rRRV vector can be found in S1 Fig. Because Ipi therapy is associated with immune-related adverse events, the rDNA-SIVnfl+Ipi vaccinations were given to two Group 2 vaccinees (Group 2a) first, before being administered to the remaining six animals (Group 2b). In order to match this immunization schedule, the RMs in Group 1 were also subdivided in Group 1a (n = 2) and Group 1b (n = 6) and vaccinated with rDNA-SIVnfl only (without any exogenous adjuvants) in parallel with their Group 2a and Group 2b counterparts. The Group 1a and Group 2a vaccinees received a second dose of the rRRV pentamix at study week 25. Because this second rRRV boost had little or no effect on vaccine-induced SIV-specific immune responses, we decided to omit it from the Group 1b and Group 2b immunization schedules. At study weeks 53–54, vaccine efficacy was assessed by subjecting all vaccinated animals and eight unvaccinated RMs (Group 3) to intrarectal (IR) challenges with a marginal dose of SIVmac239 (200 TCID_50_) every other week.

All sixteen RMs were primed four times with vectors 1 and 2. Those in Group 1 (n = 8) did not receive any exogenous adjuvant during the rDNA-SIVnfl immunizations, whereas those in Group 2 (n = 8) were infused with Ipi (3.0 mg/kg of body weight) on the day after each rDNA-SIVnfl prime (Fig 1). This rDNA-SIVnfl+Ipi priming schedule was intended to match the clinically-approved Ipi therapy regimen, which consists of four infusions of 3.0 mg/kg given every 3 weeks [12]. Six weeks after the 4^th^ priming immunization, animals in both groups were boosted with a mixture of five rRRV constructs referred to as the “rRRV pentamix” (vectors 3–7; S1C–S1G Fig; Fig 1). Three of these rRRV vectors expressed SIVnfl (vectors 3–5), albeit under the control of different promoters (S1C–S1E Fig). The SIVnfl inserts present in vectors 3–5 lacked the *tat* and *nef* modifications described above (S1C–S1E Fig). In an attempt to augment vaccine-induced anti-Env Ab responses, the rRRV pentamix also included two constructs that expressed SIV *env* alone (S1F and S1G Fig). Vector 6 encoded SIVmac239 *env* and vector 7 encoded SIVmac316 *env* (S1F and S1G Fig). Both SIV *env* inserts contained the aforementioned E_767_Stop truncation (S1F and S1G Fig).

### The rDNA-SIVnfl+Ipi immunizations are safe and immunogenic in RMs

Because Ipi therapy in humans is associated with immune-related adverse events [25], the safety of the rDNA-SIVnfl+Ipi immunizations was evaluated in two Group 2 RMs (Group 2a) first, before the remaining six Group 2 vaccinees (Group 2b) received their rDNA-SIVnfl+Ipi doses (Fig 1). For the sake of comparison, the RMs in Group 1 were also subdivided in Groups 1a (n = 2) and 1b (n = 6) and vaccinated with rDNA-SIVnfl (without any exogenous adjuvants) in parallel with their Group 2a and Group 2b counterparts. (Fig 1). The rDNA-SIVnfl+Ipi immunizations were well tolerated by the Group 2a vaccinees; neither animal experienced adverse events commonly associated with Ipi therapy (e.g., skin rashes and diarrhea) during the rDNA-SIVnfl+Ipi priming phase.

**Fig 2:**
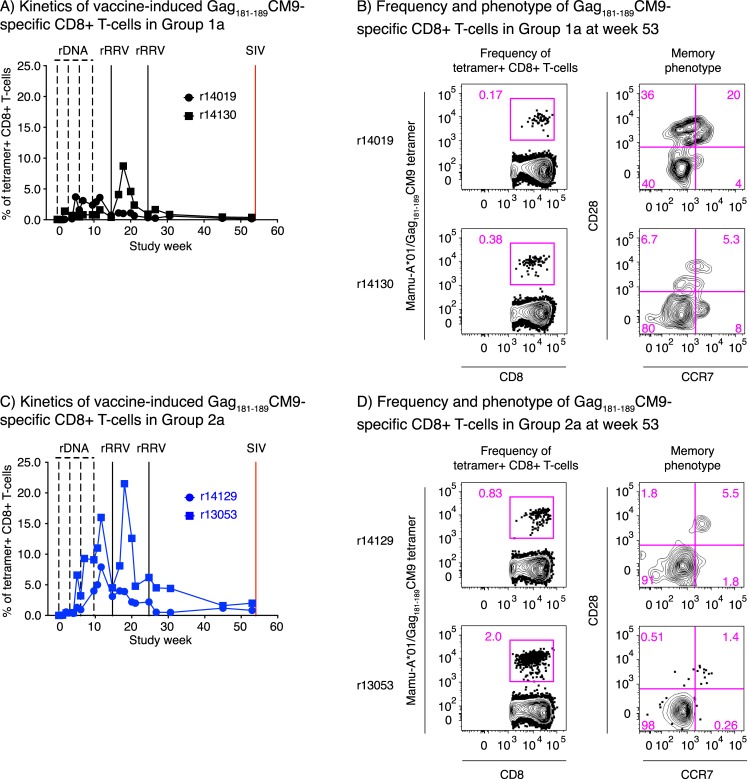
Kinetics and memory phenotype of vaccine-induced Gag_181-189_CM9-specific CD8+ T-cells. Mamu-A*01+ RMs were selected for Groups 1a and 2a to allow tracking of vaccine-induced SIV-specific CD8+ T-cells by tetramer staining. PBMC from the Group 1a (A) and Group 2a (C) vaccinees were stained with a Mamu-A*01/ Gag_181-189_CM9 tetramer at regular time points during the vaccine phase. The time scale in the x-axes matches that in Fig 1. B&D) Frequency and memory phenotype of vaccine-induced Gag_181-189_CM9-specific CD8+ T-cells in PBMC from monkeys in Group 1a (B) and Group 2a (D) at week 53. The differential expression of CD28 and CCR7 was used to delineate central memory (T_CM_; CD28+CCR7+), transitional memory (T_EM1_; CD28+CCR7–), and fully-differentiated effector memory (T_EM2_; CD28–CCR7–) subsets of tetramer+ CD8+ T-cells.

The pilot Group 1a/2a vaccinations also enabled us to track vaccine-induced SIV-specific CD8+ T-cells in peripheral blood mononuclear cells (PBMCs) by fluorochrome-labeled MHC-I tetramer staining, as the four animals in those groups expressed the MHC-I allele Mamu-A*01. Five Mamu-A*01-restricted SIV epitopes were evaluated: Vif_100-109_VL10, Env_620-628_TL9, Env_233-241_CL9, Tat_28-35_SL8, and Gag_181-189_CM9. Vaccine-elicited CD8+ T-cells directed against the first two epitopes were largely undetectable in both groups (S2A and S2B Fig). Env_233-241_CL9-specific CD8+ T-cells reached modest frequencies in the Group 2a monkey r13053, but were present at low levels in the other three vaccinees (S2C Fig). Tat_28-35_SL8-specific CD8+ T-cells remained at baseline levels during the rDNA-SIVnfl immunizations but then expanded in two animals after the first rRRV-SIVnfl boost (S2D Fig), consistent with this epitope being inactivated in the rDNA-SIVnfl constructs but intact in the rRRV pentamix. Vaccine-induced CD8+ T-cells against Gag_181-189_CM9 were detected in all four animals, especially those in Group 2a (Fig 2A). Indeed, up to 16% of peripheral CD8+ T-cells in Group 2a stained positive for the Mamu-A*01/Gag_181-189_CM9 tetramer two weeks after the 4^th^ DNA prime, compared to only 3.6% in Group 1a (Fig 2C). Boosting with rRRV-SIVnfl at week 15 further expanded these Gag-specific CD8+ T-cells, especially in the Group 2a vaccinee r13053 (Fig 2A and 2C). However, a second rRRV-SIVnfl boost given at week 25 had little or no effect on the frequencies of CD8+ T-cells targeting Gag_181-189_CM9 and the other aforementioned epitopes (Fig 2A and 2C; S2 Fig), probably as a result of anti-RRV immunity generated by the first rRRV-SIVnfl vaccination. The frequencies of vaccine-elicited Gag_181-189_CM9-specific CD8+ T-cells contracted over time in both groups but were still detectable at study week 53, the last time point before the challenge phase (Fig 2A and 2C). A memory phenotype analysis conducted at this time point revealed that the fraction of Mamu-A*01/Gag_181-189_CM9 tetramer+ CD8+ T-cells displaying a fully-differentiated effector memory T-cell (T_EM2_) signature (CD28−CCR7−) was 40–80% in Group 1a and 91–98% in Group 2a (Fig 2B and 2D). The durability of these vaccine-induced CD8+ T-cell responses and their T_EM_-biased phenotype are consistent with the ability of rRRV-SIVnfl vectors to provide chronic low-level exposure to SIV antigens.

### Immunization schedule of Groups 1b and 2b

Since the Ipi infusions were well tolerated by the Group 2a vaccinees, we proceeded with the immunization schedule of the Group 1b and Group 2b animals and delivered their first rDNA-SIVnfl or rDNA-SIVnfl+Ipi prime at study week 12 (Fig 1). Similar to the Group 1a/2a immunization schedule, the first three rDNA-SIVnfl primes were given at 3-week intervals (Fig 1). However, the 4^th^ rDNA-SIVnfl prime had to be delayed until week 24 because of an error handling the rDNA-SIVnfl plasmids (Fig 1). Again, none of the animals in Group 2b experienced Ipi-associated irAEs during the rDNA-SIVnfl+Ipi immunizations. Six weeks after the 4^th^ rDNA-SIVnfl prime, all vaccinees in Groups 1b and 2b were boosted with the rRRV pentamix (Fig 1). Since the 2^nd^ rRRV-SIVnfl administration resulted in little or no boosting of SIV-specific CD8+ T-cells in Groups 1a and 2a, we chose not to give the Group 1b and Group 2b vaccinees a second dose of the rRRV-SIVnfl pentamix (Fig 1). Even though the immunization schedules of the Groups 1a/2a and Groups 1b/2b monkeys were offset by 12 weeks, all vaccinated animals were challenged with SIVmac239 around the same time, that is, week 53 for the vaccinees in Groups 1b and 2b, and week 54 for those in Groups 1a and 2a (Fig 1).

### Pharmacokinetics of Ipi and its effects on peripheral lymphocyte counts

**Fig 3:**
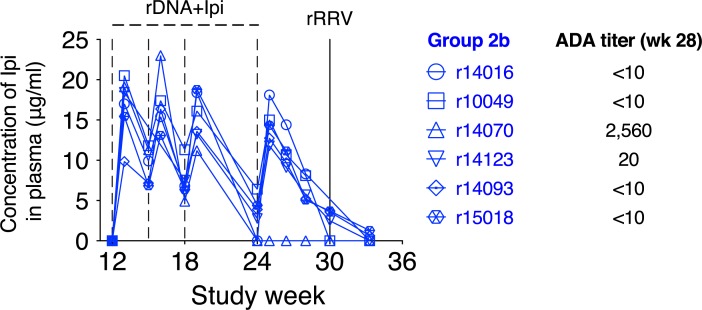
Pharmacokinetics of Ipi in the Group 2b vaccinees. The concentration of Ipi was measured in plasma from the Group 2b animals at regular intervals during the vaccine phase. Each symbol denotes one vaccinee. Anti-Ipi antibodies were quantified in plasma collected at week 28. The endpoint titer of these anti-drug antibodies (ADAs) is shown next to its corresponding animal ID. The time scale in the x-axis matches that in Fig 1.

We used ELISA to monitor the plasma concentrations of Ipi in the Group 2b vaccinees after each rDNA-SIVnfl+Ipi immunization. Ipi was readily detectable after the first rDNA-SIVnfl+Ipi immunization and its concentrations shot up after each subsequent infusion (Fig 3). Following the fourth rDNA-SIVnfl+Ipi immunization, an increase in plasma Ipi concentrations was observed in all Group 2b RMs, except for r14070 (Fig 3). The lack of detectable Ipi in r14070 was likely due to the robust anti-drug Ab (ADA) response developed by this animal. Indeed, the endpoint titer of anti-Ipi Abs in r14070 at study week 28 was 1:2,560 (Fig 3). By comparison, contemporaneous endpoint titers of ADAs were 1:20 in r14123 and less than 1:10 in the remaining Group 2b vaccinees (Fig 3). These responses were also detected in the Group 2a vaccinee r13053, whose endpoint titer of ADAs on day 14 post 4^th^ rDNA-SIVnfl+Ipi prime was 1:40.

The Ipi infusions were also associated with transient increases in the absolute counts of several peripheral lymphocyte subsets, including CD3+ T-cells, CD4+ T-cells, CD25+ FoxP3+ T regulatory cells (Tregs), CD8+ T-cells, and CD20+ B-cells (S3 Fig). Compared to Group 1b, the peripheral numbers of all these lymphocyte subsets (except for Tregs) were significantly elevated in Group 2b in at least one time point following the third rDNA-SIVnfl+Ipi priming immunization (S3 Fig).

### Anti-CTLA-4 therapy restricted the breadth of vaccine-induced SIV-specific CD8+ T-cell responses in Group 2

**Fig 4:**
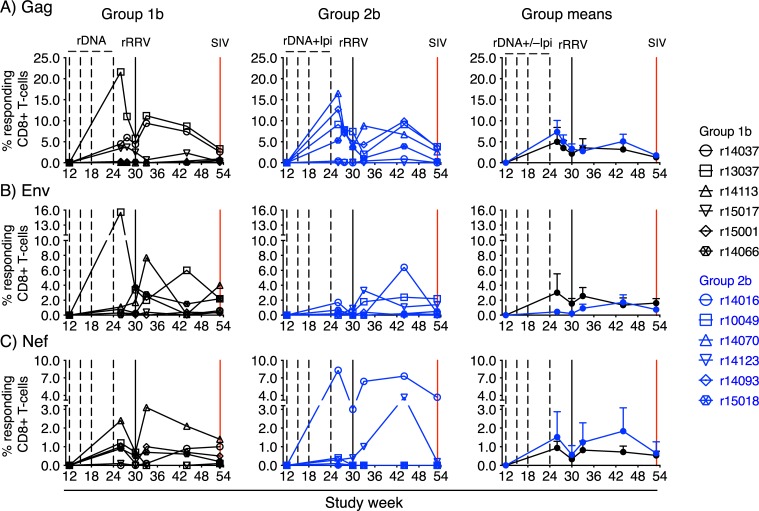
Kinetics of vaccine-induced CD8+ T-cell responses against Gag, Env, and Nef. ICS was used to quantify vaccine-induced CD8+ T-cell responses against Gag (A), Env (B), and Nef (C) in Groups 1b (left column) and 2b (middle column) at multiple time points during the vaccine phase. Group means for these responses are shown in the right column. The error bars in the right panels correspond to the standard error of the mean and each symbol in the left and middle panels denotes one vaccinee. The time scale in the x-axes matches that in Fig 1. The percentages of responding CD8+ T cells shown in the y-axes were calculated by adding the background-subtracted frequencies of positive responses producing any combination of IFN-γ, TNF-α, and CD107a. To search for differences in vaccine-induced CD8+ T-cell responses over time between Groups 1b and 2b, mixed-effect quantile regression was performed, using time and group-by-time interactions as fixed effects, and individual differences as random effects. Significant group-by-time interactions were not observed.

**Fig 5:**
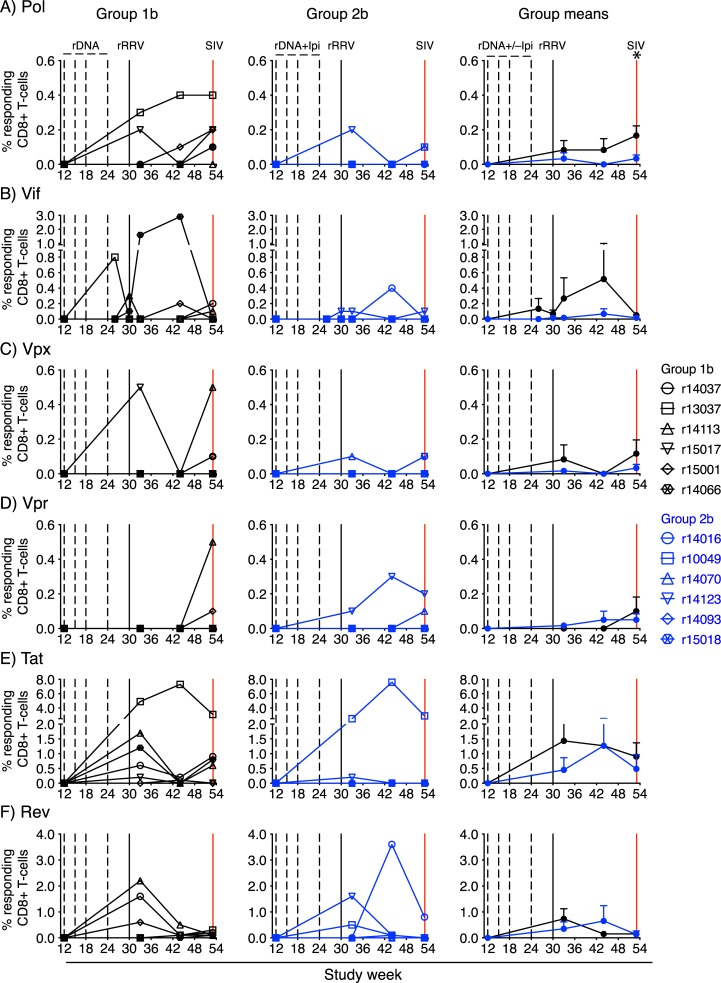
Kinetics of vaccine-induced CD8+ T-cell responses against Pol, Vif, Vpx, Vpr, Tat, and Rev. ICS was used to quantify vaccine-induced CD8+ T-cell responses against Pol (A), Vif (B), Vpx (C), Vpr (D), Tat (E), and Rev (F) in Groups 1b (left column) and 2b (middle column) at multiple time points during the vaccine phase. Group means for these responses are shown in the right column. The error bars in the right panels correspond to the standard error of the mean and each symbol in the left and middle panels denotes one vaccinee. The time scale in the x-axes matches that in Fig 1. The percentages of responding CD8+ T cells shown in the y-axes were calculated by adding the background-subtracted frequencies of positive responses producing any combination of IFN-γ, TNF-α, and CD107a. To search for differences in vaccine-induced CD8+ T-cell responses over time between Groups 1b and 2b, mixed-effect quantile regression was performed, using time and group-by-time interactions as fixed effects, and individual differences as random effects. The only instance of a significant difference in the magnitude of CD8+ T-cell responses between Groups 1b and 2b was at week 53, when Pol-specific CD8+ T-cells in Group 1b were higher than those in Group 2b. This difference is indicated by an asterisk on top right panel.

We carried out intracellular cytokine staining (ICS) at multiple time points during the vaccine phase in order to characterize vaccine-induced SIV-specific T-cell responses in Groups 1b and 2b. The stimuli for these assays consisted of pools of peptides (15mers overlapping by 11 amino acids) spanning the entire SIVmac239 proteome. Although Gag, Env, and Nef were the primary targets of vaccine-induced CD8+ T-cells in Groups 1b and 2b (Fig 4), CD8+ T-cell responses against Pol, Vif, Vpx, Vpr, Tat, and Rev were also detected (Fig 5), consistent with the ability of SIVnfl to express the entire SIV proteome. Except for a modest, but statistically significant, rise in Pol-specific CD8+ T-cells in Group 1b versus Group 2b at week 53 (Fig 5A), vaccinees in these groups developed similar levels of CD8+ T-cell responses against each SIV protein (Figs 4 and 5). CD4+ T-cell responses followed a similar pattern but were either undetectable for some of the proteins or present at much lower frequencies (S4 Fig; S5 Fig). Gag-specific CD4+ T-cells were an exception, as these responses were significantly higher in Group 2b than in Group 1b at several time points during the vaccine phase (S4A Fig). Of note, we could not detect vaccine-elicited Gag-specific T-cells in disaggregated lymphocyte suspensions from pooled colon and rectal biopsies obtained from the Group 1b and Group 2b animals at week 3 post rRRV-SIVnfl boost.

**Fig 6:**
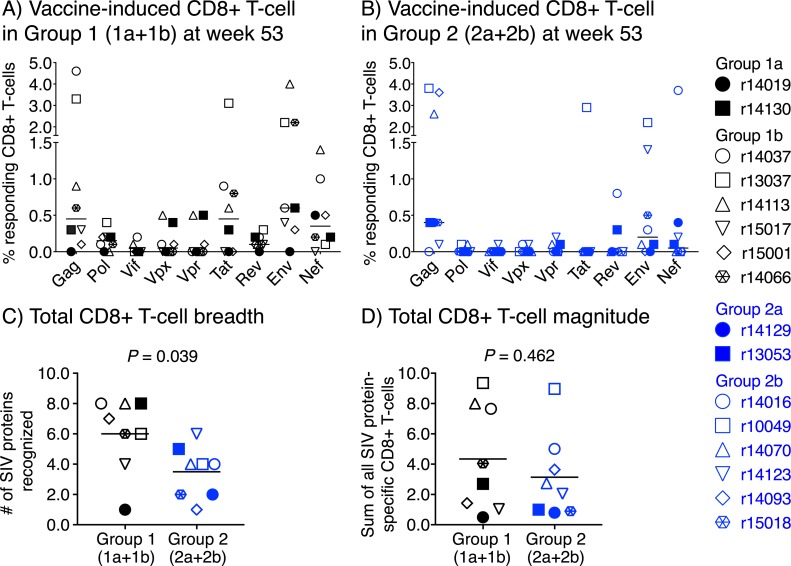
Total magnitude and breadth of vaccine-induced SIV-specific CD8+ T-cell responses at the time of SIV challenge. A&B) The frequency of vaccine-induced CD8+ T-cells targeting each of the nine SIV proteins at week 53 is shown for each animal in Group 1 (1a+1b) (A) and Group 2 (2a+2b) (B). These data were used to calculate the total breadth (i.e., sum of all SIV proteins recognized; C) and total magnitude (i.e., sum of all SIV protein-specific responses; D) of SIV-specific CD8+ T-cell responses in each animal. C&D) Comparisons of the total breadth (C) and magnitude (D) of vaccine-induced SIV-specific CD8+ T-cell responses between Groups 1 and 2. Bars correspond to means and each symbol denotes one vaccinee. *P*-values were calculated using Welch’s t-test.

We also assessed the breadth and magnitude of vaccine-elicited SIV-specific CD8+ T-cell responses at week 53, the last time point before the SIV challenge phase. The vaccinees in Groups 1a and 2a were also included in this analysis since they were challenged with SIVmac239 around the same time as their Group 1b and Group 2b counterparts. There was considerable animal-to-animal variability in the magnitude of vaccine-induced CD8+ T-cell responses targeting each protein (Fig 6A and 6B). In contrast to a previous study reporting that Ipi therapy broadens the repertoire of melanoma-reactive CD8+ T-cells in cancer patients [26], the rDNA-SIVnfl+Ipi immunizations appeared to restrict the breadth of vaccine-induced SIV-specific CD8+ T-cells in Group 2 (Fig 6C). Indeed, vaccine-elicited CD8+ T-cells in Group 2 recognized significantly fewer SIV proteins than those in Group 1 (*P* = 0.039; Fig 6C). Notwithstanding this difference, the sum of vaccine-elicited CD8+ T-cell responses against all nine SIV proteins was not significantly different between Groups 1 and 2 (Fig 6D). No significant sex differences were observed in the breadth or magnitude of vaccine-induced SIV-specific CD8+ T-cell responses within Group 1 (1a+1b) and Group 2 (2a+2b) (S6A and S6B Fig).

### Anti-CTLA-4 therapy improved binding titers but not antiviral functions of vaccine-induced Env-specific antibodies

To directly assess the impact of priming anti-Env Ab responses in the context of CTLA-4 blockade, we tracked the expansion of gp140-specific B-cells in PBMC from vaccinees in Groups 1 and 2 following the 4^th^ rDNA-SIVnfl prime. For this analysis, freshly-sorted plasmablasts were used in IgG enzyme-linked immune absorbed spot (ELISpot) assays for the detection of cells producing gp140-specific IgG. These Env-specific plasmablasts were present at low frequencies in Groups 1b and 2b on the day of the 4^th^ DNA prime, but underwent considerable expansion in the ensuing days, particularly in the Group 2b animals (S7A Fig). A similar pattern of Env-specific plasmablast expansion was observed in Groups 1a and 2a (S7B Fig). A comparison of gp140-specific plasmablast frequencies on day 7 post 4^th^ DNA prime revealed significantly higher frequencies of these cells in Group 2b than in Group 1b (S7C Fig), as well as in Group 2a than in Group 1a (S7D Fig).

**Fig 7:**
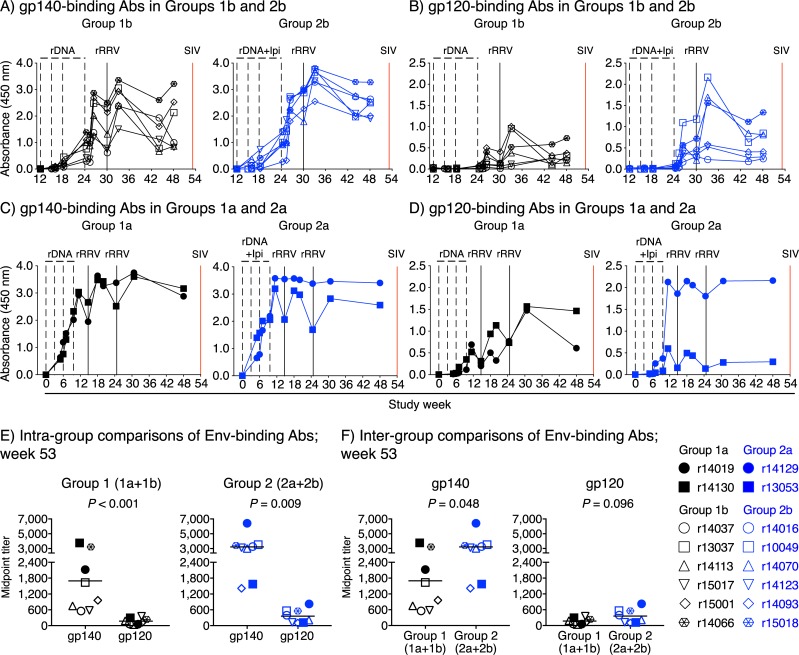
Vaccine-induced Env-binding antibody responses in Groups 1 and 2. A-D) Env-binding antibodies (Abs) were measured by ELISA using plate-bound SIVmac239 gp140 (A&C) or gp120 (B&D) at multiple time points throughout the vaccine phase. Straight 1:1,000 dilutions of plasma from each vaccinee in Groups 1b and 2b (A&B) and Groups 1a and 2a (C&D) were used for this analysis. The time scale in the x-axes matches that in Fig 1) Serial dilutions of sera collected at week 53 were used to determine the midpoint titers of Env-binding Abs in Groups 1 and 2. E) Intragroup comparisons of gp140- and gp120-binding Abs at week 53. F) Intergroup comparisons of gp140- and gp120-binding Abs at week 53. Bars correspond to means and each symbol denotes one vaccinee. *P*-values were calculated using Welch’s t-test.

A time-course analysis of vaccine-induced Env-binding Abs in Groups 1b and 2b showed that anti-gp140 Abs appeared with faster kinetics and reached greater levels in plasma than did anti-gp120 Abs (Fig 7A and 7B). These responses followed a similar pattern in Groups 1a and 2a (Fig 7C and 7D). At the time of SIV challenge, vaccinees in Group 1 (1a+1b) and Group 2 (2a+2b) had significantly higher titers of IgG Abs capable of binding gp140 than gp120 (Fig 7E), suggesting that gp41 was heavily targeted by vaccine-induced Env-specific Abs. Consistent with the plasmablast ELISpot results, this analysis also revealed that vaccinees in Group 2 (2a+2b) had greater titers of gp140-binding Abs than their Group 1 (1a+1b) counterparts (Fig 7F). However, the levels of gp120-binding Abs were not significantly different between the two groups (Fig 7F). Vaccine-induced gp140-binding Abs were also detected in rectal secretions at study week 48, that is, 5–6 weeks prior to the first SIV challenge (S8 Fig). There was no significant difference in the levels of these rectal anti-gp140 Abs between Group 1 (1a+1b) and Group 2 (2a+2b) (S8 Fig).

**Fig 8:**
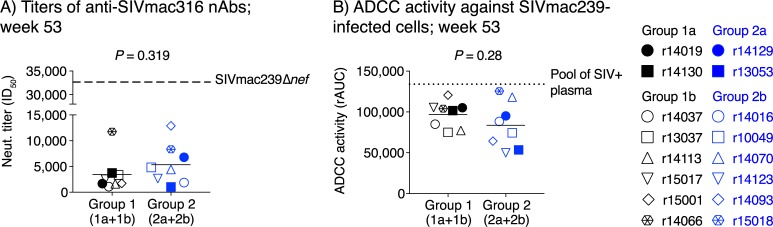
Antiviral functions of vaccine-induced anti-Env antibody responses at the time of SIV challenge. A) Neutralizing antibodies (nAbs) against SIVmac316 at week 53. Serially diluted sera from vaccinees in Group 1 (1a+1b) and Group 2 (2a+2b) were used to determine the lowest reciprocal dilution that results in 50% reduction of SIVmac316 infectivity in TZM-bl assays (ID_50_). As a reference, the mean ID_50_ titer of animals infected with SIVmac239Δ*nef* for 23 weeks is shown as a horizontal dashed line. B) NK cell-mediated Ab-dependent cellular cytotoxicity (ADCC) against SIVmac239-infected cells at week 53. Serially diluted plasma from the vaccinees in Group 1 (1a+1b) and Group 2 (2a+2b) was screened for its ability to direct a NK cell line to kill luciferase-expressing target cells infected with SIVmac239 or SHIV-AD8 (internal control for non-specific killing). Changes in relative light units under each plasma dilution were used to calculate area under curve (AUC) values for SIVmac239- and SHIV-AD8-infected cells. The area under the SHIV-AD8 curve was subtracted from the area under the SIVmac239 curve, thereby yielding a relative (r) AUC value for each animal. These rAUC values were used to compare the ADCC activity between Groups 1 and 2. As a reference, the rAUC value for pooled plasma from SIVmac239-infected RMs is shown as a horizontal dotted line. Lines correspond to mean values and each symbol denotes one vaccinee. *P*-values were calculated using Welch’s t-test.

Next, we characterized antiviral functions of vaccine-induced anti-Env Abs at the time of the first SIV challenge. While little or no serological neutralizing activity against SIVmac239 was detected, the 50% inhibitory dilution (ID_50_) titers of anti-SIVmac316 neutralizing (n)Abs in Group 1 and Group 2 ranged from 1,011 to 12,811 (Fig 8A). As a reference, these titers were 2.5- to 32-fold lower than the mean titer of anti-SIVmac316 nAbs present in sera from monkeys that had been infected with SIVmac239Δ*nef* for 23 weeks (Fig 8A). There was no significant difference in the ID_50_ titers of anti-SIVmac316 nAbs between Group 1 (1a+1b) and Group 2 (2a+2b) (Fig 8A). We also evaluated the ability of contemporaneous plasma samples to direct natural killer (NK) cell-mediated, Ab-dependent cellular cytotoxicity (ADCC) against SIVmac239-infected cells. ADCC activity was calculated based on the ability of serially diluted plasma from each animal to kill target cells infected with SIVmac239 or SHIV-AD8 (internal control). The extent that serially diluted plasma resulted in killing of target cells infected with each virus allowed us to calculate the area under the curve (AUC) for each condition. The area under the SHIV-AD8 curve was then subtracted from the area under the SIVmac239 curve, yielding a relative (r) AUC value for each animal. Based on these rAUC values, ADCC was detected in all samples analyzed and, in some cases, at levels that approximated those seen in pooled plasma from SIVmac239-infected RMs (Fig 8B). Similar to the anti-SIVmac316 nAb titers, there was no significant difference in ADCC activity between Group 1 (1a+1b) and Group 2 (2a+2b) (Fig 8B). Furthermore, no significant sex differences were observed within Group 1 (1a+1b) and Group 2 (2a+2b) in midpoint titers of Env-binding Abs, anti-SIVmac316 nAb titers, or ADCC activity (S6C–S6F Fig).

### Vaccination with rDNA/rRRV-SIVnfl, but not rDNA+Ipi/rRRV-SIVnfl, conferred significant protection against rectal acquisition of SIVmac239

Vaccine efficacy was assessed by subjecting all vaccinees in Group 1 (1a+1b) and Group 2 (2a+2b), as well as eight unvaccinated control RMs (Group 3), to repeated IR challenges with SIVmac239. Animals were exposed to a marginal dose (200 TCID_50_) of SIVmac239 every two weeks. Viral loads (VLs) determined on plasma samples collected on days 6 and 10 after each SIVmac239 exposure were used to determine which monkeys were re-challenged on day 14. Only animals that remained aviremic on days 6 and 10 after each SIV challenge were re-exposed to SIV; those with positive VLs in either sample were considered to be infected and continued to be bled at regular time points until week 12 post infection (PI).

**Fig 9:**
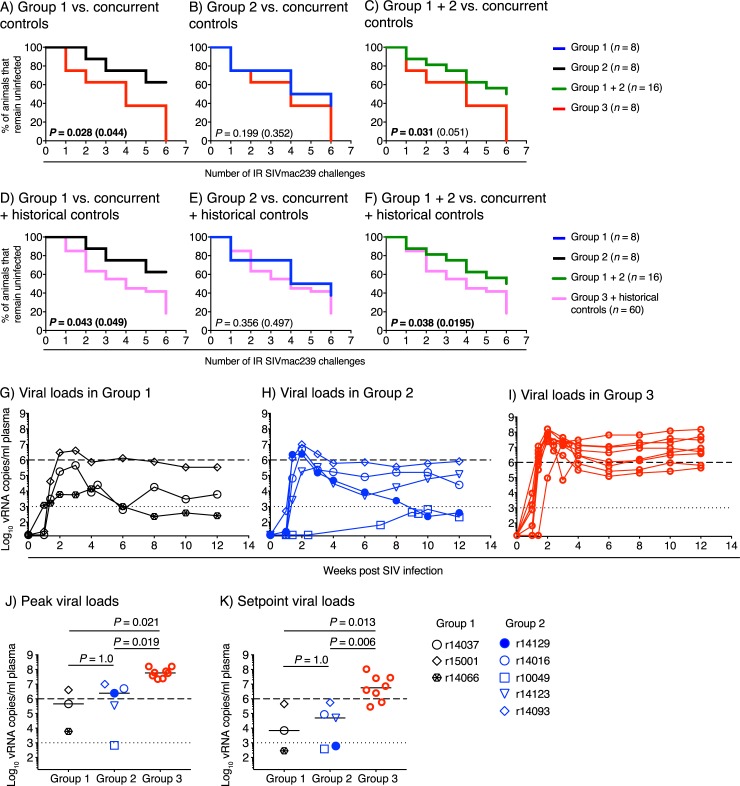
Outcome of SIV challenge. Beginning at study weeks 53–54, all vaccinees in Group 1 (1a+1b) and Group 2 (2a+2b), as well as the eight unvaccinated control RMs in Group 3, were challenged every other week with a marginal dose (200 TCID_50_) of SIVmac239 via the IR route. Animals were re-challenged only if they remained aviremic after each exposure. Since all control RMs in Group 3 acquired SIV infection by the 6^th^ challenge, the Group 1 and Group 2 vaccinees that remained uninfected at this point were no longer challenged. A-C) Kaplan-Meier analysis of SIV acquisition in vaccinees in Group 1 (n = 8; A), Group 2 (n = 8; B), and Groups 1 + 2 (n = 16; C) vs. the contemporaneous control animals in Group 3 (n = 8). D-F) Kaplan-Meier analysis of SIV acquisition in vaccinees in Group 1 (n = 8; D), Group 2 (n = 8; E), Groups 1 + 2 (n = 16; F) vs. the contemporaneous control animals in Group 3 (n = 8) plus historical control animals (n = 52). *P* values outside parentheses were determined using the Cox proportional hazard model. *P* values enclosed in parentheses were determined by Fisher’s exact test based on the number of infected and uninfected vaccinees versus controls after six SIV challenges. *P* values below 0.05 are in boldface type. G-I) Viral load (VL) kinetics in the SIV-infected RMs in Group 1 (G), Group 2 (H), and Group 3 (I). J&K) Comparison of peak (J) and setpoint (K) VLs between Group 3 and each vaccinated group. Peak VLs were determined as the highest VL measurement within the first 4 weeks after infection. Because of the unusually delayed VL kinetics observed in the Group 2 vaccinee r10049, its highest VL measurement at week 10 post infection (670 vRNA copies/ml) was considered as the peak VL for this animal. Setpoint VLs were calculated as the geometric mean of all VLs measured between weeks 8 and 12 post infection. Lines correspond to median values and each symbol denotes one vaccinee. ANOVA was used to search for group differences in log_10_-transformed peak and setpoint VLs. Significant group differences were found, so post-hoc pairwise comparisons were performed using Welch’s t test. The dotted lines in panels G-K are for reference only and indicate a VL of 10^3^ vRNA copies/ml of plasma. The dashed lines in those panels are also for reference only and denote a VL of 10^6^ vRNA copies/ml of plasma.

Following six IR challenges with SIVmac239, all eight control animals in Group 3 became infected, compared to only three vaccinees in Group 1 and five vaccinees in Group 2. This difference was statistically significant for Group 1 (*P* = 0.044, Fisher’s exact test) but not for Group 2 (*P* = 0.352, Fisher’s exact test). The rate of SIV acquisition in Group 1 was also significantly slower than that in Group 3 (*P* = 0.028, Cox proportional hazard model; Fig 9A), indicating that the rDNA/rRRV-SIVnfl vaccine regimen afforded significant protection against IR challenge with SIVmac239. Specifically, vaccination with the rDNA/rRRV-SIVnfl regimen reduced the per-exposure probability of SIV infection by 78% in Group 1. In contrast, the rDNA+Ipi/rRRV-SIVnfl vaccine regimen did not confer significant protection against SIVmac239 acquisition, as the pace of SIV infection in Group 2 was not significantly different than that in Group 3 (*P* = 0.199, Cox proportional hazard model; Fig 9B). The per-exposure probability of infection in Group 2 was reduced by 52% but it was not significantly different than that in the control group. Since there was no significant difference in infection rates between Groups 1 and 2 (*P* = 0.506, Cox proportional hazard model), we asked whether a vaccine-mediated effect on SIV acquisition could still be detected if all 16 vaccinees in Groups 1 and 2 were grouped together and compared to Group 3. This analysis revealed that the pace of SIV infection in all 16 vaccinees (Groups 1 + 2) was still significantly slower than that of the control group (*P* = 0.031, Cox proportional hazard model; Fig 9C).

In order to validate these findings, we compared the rates of SIV acquisition in Group 1, Group 2, and Groups 1 + 2 to that of an expanded control group consisting of the 8 contemporaneous RMs in Group 3 plus 52 historical control RMs (i.e., 60 control animals total). These historical RMs were part of previous experiments conducted by our group and were subjected to the same IR challenge regimen described above, utilizing the same dose of the same stock of SIVmac239 used here. This analysis confirmed the findings described above, with partial (albeit statistically significant) vaccine-mediated protection being detected in Group 1 (*P* = 0.043, Cox proportional hazard model; Fig 9D) and Group 1 + 2 (*P* = 0.038, Cox proportional hazard model; Fig 9F), but not in Group 2 (*P* = 0.356, Cox proportional hazard model; Fig 9E). Of note, immunological measurements performed in the eight vaccinees that remained uninfected two weeks after the 6^th^ SIV exposure revealed no significant enhancement in vaccine-induced SIV-specific immune responses compared to the levels measured just prior to the first SIV challenge (S9 Fig), thereby confirming their protected status.

Vaccine-mediated reductions in viremia were evident by visual inspection of the VL profiles of control and vaccinated RMs (Fig 9G–9I). While there was no difference in peak and setpoint VLs between the SIV-infected vaccinees in Groups 1 and 2, their VLs were significantly lower than those in the Group 3 controls (Fig 9J and 9K).

### Conventional measurements of vaccine-induced SIV-specific immune responses did not predict SIV challenge outcome

Lastly, we searched for immune attributes that could distinguish the vaccinees that were protected from SIVmac239 infection versus those that were not. We selected 7 pre-challenge attributes of vaccine-induced immune responses for this analysis: i) the total breadth of SIV-specific CD8+ T-cell responses; ii) the total magnitude of SIV-specific CD8+ T-cell responses; iii) the serum midpoint titers of gp140- and (iv) gp120-binding IgG Abs; (v) the serum titers of anti-SIVmac316 nAbs; (vi) the levels of ADCC activity against SIVmac239-infected cells (rAUC values); and the (vii) rectal levels of gp140-binding IgG Abs. All vaccinees in Groups 1 and 2 were included in this analysis. None of these immunological variables were associated with resistance to SIVmac239 infection (Table 1). We also searched for correlations between these immunological variables and either peak or setpoint VLs in the vaccinees that acquired SIV infection, but again found no significant association (Table 2).

**Table 1:** Lack of association between pre-challenge attributes of vaccine-induced immune responses in Groups 1 and 2 and acquisition of SIV infection^1^.

Immunological variable	Hazard ratio (95% confidence interval)	*P*-value
Total breadth of SIV-specific CD8+ T-cell responses^2^	0.996 (0.457, 2.169)	0.992
Total magnitude of SIV-specific CD8+ T-cell responses^3^	1.061 (0.553, 2.038)	0.858
Serum midpoint titers of gp140-binding IgG Abs^4^	1.785 (0.763, 4.178)	0.182
Serum midpoint titers of gp120-binding IgG Abs^4^	1.451 (0.687, 3.064)	0.329
Serum titers of anti-SIVmac316 nAbs^5^	1.484 (0.765, 2.879)	0.243
ADCC activity against SIVmac239-infected cells^6^	0.799 (0.395, 1.617)	0.533
Rectal levels of gp140-binding IgG Abs^7^	0.855 (0.393, 1.862)	0.693

^1^ To appropriately account for censoring (five animals in Group 1 and three in Group 2 did not acquire SIV infection), the Cox proportional hazard model was used for this analysis. As the scale of immunological responses varies, the values are standardized.

^2^ Based on the data shown in Fig 6C.

^3^ Based on the data shown in Fig 6D.

^4^ Based on the data shown in Fig 7E and 7F.

^5^ Based on the data shown in Fig 8A.

^6^ Based on the data shown in Fig 8B.

^7^ Based on the data shown in S8 Fig.

**Table 2:** Lack of association between pre-challenge attributes of vaccine-induced immune responses in Groups 1 and 2 and either peak or setpoint VLs^1^.

**Peak VLs**	**Immunological variable**	**Correlation coefficient**	***P*-value**
	Total breadth of SIV-specific CD8+ T-cell responses^2^	–0.301	0.468
	Total magnitude of SIV-specific CD8+ T-cell responses^3^	–0.381	0.360
	Serum midpoint titers of gp140-binding IgG Abs^4^	–0.262	0.536
	Serum midpoint titers of gp120-binding IgG Abs^4^	–0.238	0.582
	Serum titers of anti-SIVmac316 nAbs^5^	0.000	1.000
	ADCC activity against SIVmac239-infected cells^6^	0.071	0.882
	Rectal levels of gp140-binding IgG Abs^7^	–0.036	0.933
**Setpoint VLs**	**Immunological variable**	**Correlation coefficient**	***P*-value**
	Total breadth of SIV-specific CD8+ T-cell responses^2^	–0.012	0.977
	Total magnitude of SIV-specific CD8+ T-cell responses^3^	–0.310	0.462
	Serum midpoint titers of gp140-binding IgG Abs^4^	–0.500	0.216
	Serum midpoint titers of gp120-binding IgG Abs^4^	–0.476	0.243
	Serum titers of anti-SIVmac316 nAbs^5^	0.167	0.703
	ADCC activity against SIVmac239-infected cells^6^	–0.190	0.665
	Rectal levels of gp140-binding IgG Abs^7^	–0.395	0.333

^1^ This analysis included only the SIV-infected vaccinees in Group 1 (n = 3) and Group 2 (n = 5). The Spearman rank correlation was used to search for associations between the aforementioned immunological variables and plasma viral loads.

^2^ Based on the data shown in Fig 6C.

^3^ Based on the data shown in Fig 6D.

^4^ Based on the data shown in Fig 7E and 7F.

^5^ Based on the data shown in Fig 8A.

^6^ Based on the data shown in Fig 8B.

^7^ Based on the data shown in S8 Fig.

**Fig 10:**
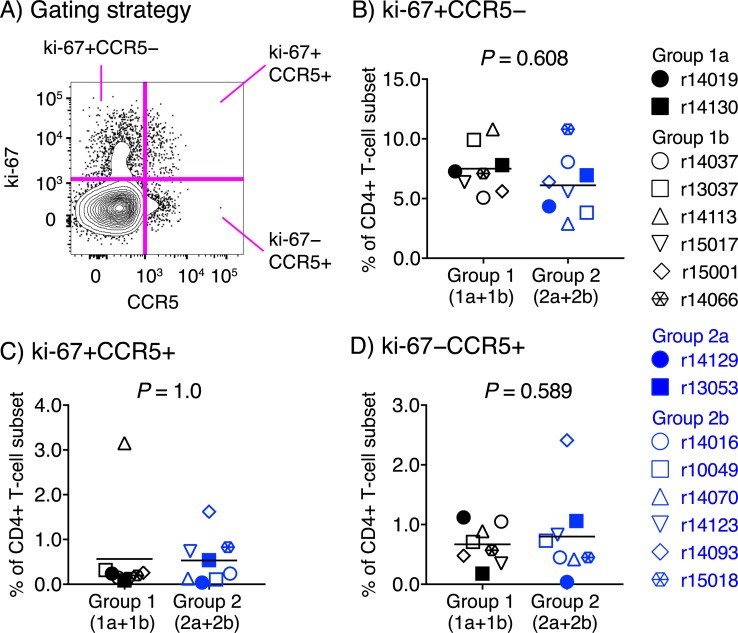
Pre-challenge frequencies of activated CD4+ T-cells in Groups 1 and 2. Cryopreserved PBMC samples from the Group 1 (1a+1b) and Group 2 (2a+2b) vaccinees collected at study week 50 were thawed and used in flow cytometric assays to assess the expression of ki-67 and/or CCR5 on CD4+ T-cells. A) Gating strategy. Cells were gated on live CD14− CD16− CD20− CD3+ CD4+ CD8− lymphocytes. B-D) Comparisons of the frequencies of ki-67+CCR5−CD4+ T-cells (B), ki-67+CCR5+CD4+ T-cells (C), and ki-67−CCR5+CD4+ T-cells (D) between Groups 1 and 2. Lines correspond to mean values and each symbol denotes one vaccinee. *P*-values were calculated using median regression (see the Statistics section in Materials and Methods).

Since one of the hallmarks of anti-CTLA-4 therapy in humans is the expansion of effector-like CD4+ T-cells [27], we also investigated whether the Ipi infusions could have undermined the efficacy of the Group 2 vaccine regimen by increasing the availability of SIV target cells prior to the first SIVmac239 challenge. To do that, we compared the pre-challenge frequencies of peripheral CD4+ T-cells expressing ki-67 and/or CCR5 between Groups 1 and 2 (Fig 10A). This analysis revealed that the proportions of ki-67+CCR5− (Fig 10B), ki-67+CCR5+ (Fig 10C), and ki-67−CCR5+ (Fig 10D) CD4+ T-cell subsets in PBMC were not significantly different between Groups 1 and 2, suggesting that increased SIV target cell availability did not underlie the failure of the Group 2 vaccine regimen to protect against SIV infection. Thus, the immunological basis by which the rDNA/rRRV-SIVnfl vaccine regimen protected a subset of Group 1 animals against rectal acquisition of SIVmac239 remains unclear.

## Discussion

Experimental challenge of RMs with SIV provides a valuable system for identifying immune correlates of protection and selecting the most promising vaccine approaches for human testing [28]. Because a successful outcome in this model can be used to justify costly clinical trials, it is critical that the efficacy of pre-clinical AIDS vaccine concepts be assessed against stringent challenge viruses. The SIVmac239 molecular clone is well suited for this purpose. Consistent with its neutralization resistance and high replicative capacity in Indian-origin RMs, vaccine protection against SIVmac239 acquisition is exceedingly difficult to achieve, even when there is a complete match between vaccine-encoded sequences and the challenge virus. Several vaccine trials have reported significant reductions in post-acquisition SIVmac239 viremia in RMs but no protection from infection [29–34]. Even the rhesus cytomegalovirus-based vaccine platform developed by Picker and colleagues, which results in profound control and eventual clearance of SIVmac239 infection in half of vaccinees [35–37], does not block acquisition of SIVmac239. In fact, live-attenuated strains of SIVmac239 (e.g., SIVmac239Δ*nef*) remain the only vaccine modality to consistently afford significant levels of apparent sterilizing immunity against SIVmac239 challenge in Indian-origin RMs [38–40]. Given the stringency of this challenge model, it is remarkable that following repeated IR challenges with SIVmac239, the Group 1 (rDNA/rRRV-SIVnfl) vaccine regimen significantly decreased the per-exposure probability of infection by 78%. This result is reinforced by our recent demonstration that delivery of the same vectors in reverse order (i.e., rRRV-SIVnfl prime/rDNA-SIVnfl boost) also significantly reduced the per-exposure probability of IV SIVmac239 infection by 79% in RMs [18]. While the mechanisms of this protection remain unknown, we posit that the use of SIVnfl to prime and, in the case of the rRRV-SIVnfl vectors, continually boost SIV-specific immune responses facilitated the induction of protective anti-SIVmac239 immunity. From the standpoint of HIV vaccination, immunization with near full-length (nfl) viral genomes has both practical and biological advantages. For example, delivering the entire HIV proteome in a single rDNA plasmid or viral vector, as opposed to different genes in separate constructs, would likely reduce manufacturing costs and face fewer regulatory barriers down the translational pipeline. Additionally, since B-cells tend to respond with high avidity to virus-sized antigens [41], a properly adjuvanted HIVnfl-based vaccine could be expected to elicit high titers of anti-Env Ab responses, especially if this vaccine contains modifications to stabilize Env trimers and increase their surface expression [42]. Furthermore, based on the ability of the SIVnfl insert to induce CD8+ T-cell responses against the entire SIV proteome, an HIVnfl-based vaccine could also be useful for generating broadly-targeted HIV-specific CD8+ T-cell responses, thereby providing an extra line of defense in the event of HIV transmission.

We also evaluated whether treating RMs with the CTLA-4-blocking mAb Ipi during antigen priming could enhance the SIV-specific immune response induced by the rDNA/rRRV-SIVnfl vaccine regimen. In contrast to the powerful immunological effects of anti-CTLA-4 therapy in humans [12], the impact of the rDNA-SIVnfl+Ipi priming immunizations on vaccine immunogenicity was modest and, at least in one case, detrimental. Although the Ipi infusions likely contributed to the moderately elevated CD4+ T-cell responses and Env-binding Abs detected in Group 2, the anti-CTLA-4 therapy was also associated with a narrower repertoire of vaccine-induced SIV-specific CD8+ T-cell responses in Group 2 compared to Group 1. We propose two explanations for this worse-than-expected adjuvant activity of Ipi therapy. The first one relates to the dose of Ipi. The Group 2 vaccinees received a series of four infusions of 3.0 mg/kg/dose of Ipi, a regimen that was clinically approved based on its ability to enhance anti-tumor immunosurveillance in patients with advanced melanoma [12]. This dose might not, however, be sufficient to amplify pathogen-specific immune responses induced by prophylactic vaccination. While previous studies have reported increased vaccine- or infection-induced SIV-specific T-cell responses in nonhuman primates treated with 10.0 mg/kg/dose of Ipi [15, 16, 43], rashes or death were reported in two of those studies [16, 44], suggesting that any gains in vaccine immunogenicity achieved by raising the dose of Ipi might be counterbalanced by an increased incidence of immune-related adverse events. The second possibility is that ADAs triggered by the Ipi infusions interfered with the ability of this mAb to block CTLA-4 *in vivo*. Consistent with this idea, ADAs were detected in 3/8 Group 2 vaccinees (~38%) and appeared to accelerate the clearance of Ipi in r14070, the monkey with the highest ADA response. The fact that clinical grade Ipi (a human IgG1 molecule) was used in this study likely favored the induction of ADAs in Group 2. It is noteworthy, however, that Ipi-treated cancer patients also develop ADAs at a rate (26%) that is not too distant than the one observed here [45]. Thus, ADAs might compromise the efficacy of mAb-based therapies even when there is complete species match between the mAb and its recipients.

Although CTLA-4 is known to regulate the primary immune response to antigen exposure, there is still no consensus on whether Ipi therapy enhances anti-tumor immunosurveillance by boosting pre-existing neoantigen-specific CD8+ T-cells or priming CD8+ T-cells against new tumor epitopes [26, 46, 47]. We actually used rDNA-SIVnfl+Ipi to boost SIV-specific immune responses in the rRRV-SIVnfl-primed RMs in our recent SIV vaccine trial [18]. Those animals mounted robust anamnestic SIV-specific cellular and humoral immune responses following the rDNA-SIVnfl+Ipi boosters and were ultimately protected against IV challenges with SIVmac239. However, because of the lack of a group of Ipi-untreated, rRRV-SIVnfl/rDNA-SIVnfl-vaccinated RMs in that study, the precise contribution of anti-CTLA-4 therapy to the efficacy of the rRRV-SIVnfl/rDNA-SIVnfl vaccine regimen is unknown. Since CTLA-4 has been shown to have distinct roles in the regulation of naïve and memory T-cells [48–51], it is possible that anti-CTLA-4 therapy might have had greater adjuvant activity had it been co-administered with rDNA-SIVnfl booster immunizations.

In sum, here we show that a rDNA-SIVnfl/rRRV-SIVnfl vaccine regimen provided significant, albeit partial, protection against rectal acquisition of SIVmac239 in RMs. No protection was observed in a group of RMs vaccinated in parallel with the same immunization protocol in which infusions of the CTLA-4-blocking mAb Ipi were given after each rDNA-SIVnfl immunization. Future studies should focus on the identification of the immune responses that are critical for the antiviral properties of SIVnfl vaccination and whether SIVnfl vaccine efficacy can be improved by methods other than anti-CTLA-4 adjuvant therapy.

## Materials and methods

### Ethics statement

Twenty-four RMs (*Macaca mulatta*) of Indian origin were used in this study. All animals were housed at the Wisconsin National Primate Research Center (WNPRC) and cared for under a protocol approved by the University of Wisconsin Graduate School Animal Care and Use Committee (animal welfare assurance no. A3368-01; protocol no. G005563). The macaques in this study were managed according to the animal husbandry program of the WNPRC, the guidelines of the Weatherall Report and the principles described in the National Research Council’s Guide for the Care and Use of Laboratory Animals. The animal husbandry program of the WNPRC aims at providing consistent and excellent care to nonhuman primates at the center. This program is employed by the Colony Management Unit and is based on the laws, regulations, and guidelines promulgated by the United States Department of Agriculture (e.g., the Animal Welfare Act and its regulations, and the Animal Care Policy Manual), Institute for Laboratory Animal Research (e.g., Guide for the Care and Use of Laboratory Animals, 8th edition), Public Health Service, National Research Council, Centers for Disease Control, and the Association for Assessment and Accreditation of Laboratory Animal Care International. The nutritional plan utilized by the WNPRC is based on recommendations published by the National Research Council. Specifically, macaques were fed twice daily with 2050 Teklad Global 20% Protein Primate Diet and food intake was closely monitored by Animal Research Technicians. This diet was also supplemented with a variety of fruits, vegetables, and other edible objects as part of the environmental enrichment program established by the Behavioral Management Unit. Paired/grouped animals exhibiting stereotypical and/or incompatible behaviors were reported to the Behavioral Management staff and managed accordingly. All primary enclosures (i.e., stationary cages, mobile racks, and pens) and animal rooms were cleaned daily with water and sanitized at least once every two weeks. Lights were on a 12:12 diurnal schedule. Vaccinations were performed under anesthesia (Ketamine administered at 5–12 mg/kg depending on the animal) and all efforts were made to minimize suffering. Euthanasia was performed whenever an animal experienced conditions deemed distressful by one of the veterinarians at the WNPRC. All euthanasia were performed in accordance with the recommendations of the Panel on Euthanasia of the American Veterinary Medical Association and consisted of an IV overdose (greater than or equal to 50 mg/kg or to effect) of sodium pentobarbital or equivalent, as approved by a clinical veterinarian, preceded by ketamine (at least 15 mg/kg body weight) given by the IM route. The MHC-I genotype, sex, and age of each monkey at the beginning of the SIV challenge phase are shown in Table 3. All animals in Groups 1 and 2 were RRV seronegative in the beginning of the vaccine phase.

**Table 3:** Animal characteristics.

Experimental Group	Animal ID	Relevant MHC-I allele	Age (yrs)	Sex
1a	r14019	Mamu-A*01	4.0	Male
1a	r14130	Mamu-A*01	3.4	Female
1b	r14113	Mamu-A*01	3.5	Male
1b	r14037		4.0	Female
1b	r13037		4.9	Female
1b	r15017		3.0	Male
1b	r15001		3.3	Male
1b	r14066		3.8	Male
2a	r14129	Mamu-A*01	3.4	Female
2a	r13053	Mamu-A*01	4.8	Female
2b	r14016		4.0	Female
2b	r10049		7.9	Female
2b	r14070		3.7	Male
2b	r14123		3.4	Male
2b	r14093		3.6	Male
2b	r15018		3.0	Male
3	rh2471	Mamu-A*01	9.0	Female
3	r13057	Mamu-A*01	4.8	Female
3	r10017		8.2	Male
3	r12052		5.8	Male
3	r13034		4.9	Male
3	r13049		4.8	Male
3	r14007		4.3	Male
3	r15049		2.6	Female

### Vaccinations

The RMs in Groups 1 and 2 were primed four times with vectors 1 and 2 (S1 Fig), which utilized the pCMVkan backbone. These rDNA vectors contained SIVnfl inserts that differed in the *env* genes they encoded (S1 Fig). Each animal was vaccinated with 2.0 mg of each rDNA-SIVnfl vector per occasion (total: 4.0 mg per occasion). This dose was split into two separate syringes, so that a total of four syringes, each containing 1.0 mg of rDNA-SIVnfl, were used to deliver vectors 1 and 2 in each immunization. PBS was used to adjust the volume of each syringe to 0.5 ml. These rDNA-SIVnfl formulations were administered intramuscularly by the TriGrid *in vivo* electroporation system (Ichor Medical Systems, Inc., San Diego, CA). Muscles in the thighs and forearms were used for these vaccinations. On the day after each rDNA-SIVnfl immunization, the Group 1 vaccinees were treated with 3.0 mg/kg of clinical grade Ipilimumab (Bristol-Myers Squibb). After the second rDNA-SIVnfl immunization, the Ipi infusion that was supposed to be given to r14129 (Group 2a) was, instead, given to r14019 (Group 1a) by mistake.

Six weeks after the 4^th^ rDNA-SIVnfl prime, all animals in Groups 1 and 2 were boosted with a mixture of five rRRV vectors (termed “rRRV pentamix”). A dose of 1.0×10^10^ genome copies of each rRRV vector was administered intravenously in 1.0 ml of PBS. Three of the vectors in the rRRV pentamix expressed SIVnfl, albeit under the control of different promoters. Vector 3 contained the CMV enhancer/promoter (pCMV) placed upstream of the SIVnfl insert (S1 Fig). In vector 4, a hybrid early/late promoter construct consisting of the late promoter for RRV ORF26 (p26) and the early promoter for the RRV Poly Adenylated Nuclear RNA (PAN) was inserted just upstream of the SIVnfl insert (S1 Fig). This artificial promoter was termed pDUAL (S1 Fig). In vector 5, the SIV promoter/enhancer region was used by restoring nucleotides 1–521 of the 5’ long terminal repeat (pLTR) that had been deleted in the original SIVnfl insert (S1 Fig). We used this combination of three promoters because the extent to which one may be better than another in terms of the ability to express *in vivo* in monkeys, the ability to persist, and the magnitude, quality, and persistence of resultant immune responses are unknown. We therefore hoped that these three promoters would promote stable expression of SIVnfl during all stages of the RRV life cycle. In order to maximize the generation of anti-Env Abs, the rRRV pentamix also contained two additional constructs that encoded SIV *env* alone. Vector 6 encoded SIVmac239 *env* and vector 7 encoded SIVmac316 *env* (S1 Fig). Both vectors expressed a truncated version of SIV Env (E_767_Stop) intended to increase Env incorporation into non-infectious SIV virions [19]. The codon usage of these *env* inserts was modified to reflect the codon usage of RRV glycoprotein in order to allow adequate expression in the monkeys [52]. The monkeys in Groups 1a and 2a received a second dose of the rRRV pentamix at study 25, that is, ten weeks after the first rRRV pentamix boost. However, since this second rRRV boost resulted in little or no expansion of SIV-specific immune responses, we decided to omit it in the Group 1b and Group 2b immunization schedules.

### SIVmac239 challenges

At study weeks 53 or 54 (Fig 1), all vaccinated and control RMs were subjected to repeated IR inoculations of 200 TCID_50_ (4.8×10^5^ vRNA copies) of SIVmac239. This challenge inoculum was administered in 1.0 ml of PBS. IR challenges occurred every two weeks. Plasma VLs were assessed six and ten days after each exposure. Once an animal experienced a positive VL at either one of these time points, it was no longer challenged. Only RMs that remained aviremic at both time points were re-challenged on day 14.

### SIV RNA viral load measurements

VLs were measured using 0.5 ml of EDTA-anticoagulated RM plasma based on a modification of a previously published [53]. Total RNA was extracted from plasma samples using QIAgen DSP virus/pathogen Midi kits, on a QIASymphonyXP laboratory automation instrument platform. Six replicate two-step RT-PCR reactions were performed per sample using a random primed reverse transcription reaction, followed by 45 cycles of PCR using the following primers and probe: forward primer: SGAG21: 5’-GTCTGCGTCAT(dP)TGGTGCA TTC-3’; reverse primer SGAG22: 5’-CACTAG(dK)TGTCTCTGCACTAT(dP)TGTTTTG-3’; probe: PSGAG23: 5’-FAM-CTTC(dP)TCAGT(dK)TGTTTCACTTTCTCTTCTGCG-BHQ1- 3’. The limit of reliable quantitation on an input volume of 0.5 ml of plasma was 15 vRNA copies/ml.

### Sample processing

We isolated peripheral blood mononuclear cells (PBMC) from EDTA-treated blood by Ficoll-Paque Plus (GE Health Sciences) density centrifugation. Cells were subsequently washed in R10 medium [RPMI 1640 medium supplemented with GlutaMAX (Life Technologies), 10% FBS, and 1% antibiotic/antimycotic] and then resuspended at various concentrations depending on the application.

In order to isolate lymphocytes from rectal and colon biopsies from the same animal, these specimens were pooled and resuspended in 10 mls of RPMI 1640 medium supplemented with GlutaMAX (Life Technologies) containing Liberase (Sigma-Aldrich; 40 μg/ml), DNase I (Akron Biotechnology; 4.0 μg/ml), and 1% antibiotic/antimycotic. Conical tubes containing these samples were rocked at 200 rpm for 1 hour (hr) at 37°C on a table-top shaker. At the end of this step, the cell suspensions were passed through 70-μm cell strainers, washed once in R10, and then used in immunological assays.

Weck-cel sponges containing rectal secretions were frozen at −80°C on the day of collection. Prior to use in immunological assays, these sponges were thawed and eluted using a fresh mixture of Protease Inhibitor Cocktail (P8340; Sigma-Aldrich), PBS with 0.25% bovine serum albumin, and Igepal (Sigma-Aldrich). Sponges were first spun using Spin-X columns (0.22-μm filters) and then concentrated with Vivaspin protein concentrator columns (50 kDa, 500 μl, GE Healthcare Life Sciences). These rectal secretions were eluted in a final volume of ~90 μl.

### Enzyme-linked immunosorbent assay (ELISA) measurements

The kinetics of anti-Env Abs during the vaccine phase were measured by a semi-quantitative ELISA. To begin, ELISA plates were coated with 100 μl of purified SIVmac239 gp140 or gp120 protein (Immune Technology Corp.) at a concentration of 1.0 μg/ml. These plates were incubated overnight at room temperature. On the following day, the plates were washed with PBS-Tween20 and wells were blocked with 300 μl of 5% powdered milk in PBS for 1 hr at 37°C. Subsequently, the plates were washed and plasma samples diluted 1:1,000 were added in 100 μl to the corresponding wells. After a 1-hr incubation at room temperature, the plates were washed and 100 μl of a 1:10,000 dilution of goat anti-human IgG HRP-conjugated detection antibody (Southern Biotech) was added to all wells and incubated for 1 hr at 37°C. Finally, the plates were washed before being developed with 100 μl of 3,3',5,5'-Tetramethylbenzidine (EMD Millipore). After a short incubation, the reaction was stopped with TMB Stop Solution (Southern Biotech) and the plates were read (Biotek Synergy 2) at 450 nm. The same ELISA setup was used to determine the midpoint titers of anti-gp140 and anti-gp120 Abs, except that the biological specimen used for this analysis was serially diluted serum collected at the time of the first SIV challenge. The midpoint titers of anti-Env Abs were determined using the “Sigmoidal, 4PL, X is log(concentration)” function in Prism (version 7.0e, GraphPad Software, Inc.).

The relative levels of anti-gp140 Abs in rectal secretions were determined by coating half-well ELISA plates (Corning Inc.) overnight at 4°C with either SIVmac239 gp140 (2.0 μg/ml) or purified mouse anti-human IgG (5.0 μg/ml). The plates were then washed and blocked with powdered milk as described above. After an additional wash, serial dilutions of the eluted fractions of the rectal secretions were added to the plates. To do these serial dilutions, the 85-μl eluate from each rectal weck was diluted 1:3 followed by serial 3-fold dilutions. Rectal wecks from animals chronically infected with SIVmac239 were included as internal controls. The rhesus macaque IgG1 mAb 5L7 and purified rhesus IgG, both produced in house, were used as standards at a starting concentration of 2.0 μg/ml, followed by 4-fold serial dilutions. After a 1-hr incubation at room temperature, the plates were washed and the same detection antibody described above was added to the plates for 1 hr at 37°C. The plates were developed and read as described above. The concentrations of anti-gp140 Abs in rectal secretions were normalized to the total IgG content in each sample.

The pharmacokinetics of Ipi was determined in plasma from the Group 2 animals. Half-well ELISA plates (Corning Inc.) were coated overnight at 4°C with 1.0 μg/ml of a chimeric CTLA-4-Ig protein consisting of human CTLA-4 (CD152) and a mouse IgG2a Fc tag at its C-terminus (Acro Biosystems). The plates were washed with PBS-Tween20 and blocked with 5% bovine serum albumin (BSA; Spectrum Chemical) in PBS for 1 hr at 37°C. The plates were then washed with PBS-Tween20 and the serially diluted plasma samples were added. The same Ipi batch (Bristol-Myers Squibb) that was infused into the Group 2 animals was used as the standard for this assay. The starting concentration of the Ipi standard was 5.0 μg/ml, which was serially diluted 2-fold. Bound antibodies were tagged with a goat anti-human IgG HRP-conjugated detection antibody (1:10,000 dilution; Southern Biotech) and incubated for 1 hr at 37°C. The plates were developed and read as described above. The concentration of Ipi in plasma was determined using the “Sigmoidal, 4PL, X is log(concentration)” function in Prism (version 7.0e, GraphPad Software, Inc.).

Anti-Ipi antibodies were quantified by coating half-well ELISA plates with 50 μl of Ipilimumab (Bristol Myers-Squibb) at a concentration of 10.0 μg/ml. These plates were covered with a plastic film and incubated overnight at 4°C. On the next day, the plates were washed with 1x PBS-Tween20 and blocked with 150 μl of Superblock reagent (Thermo Fischer) in PBS for 15 min at room temperature. The plates were washed again, and 50 μl of 2-fold serially diluted plasma was added to the corresponding wells. The plates were incubated for 1 hr at room temperature and then washed. Next, 50 μl of Anti-Ig human lambda light chain-biotin (Miltenyi Biotec, Inc.) diluted 1:100 in dilution buffer was added to each well. Plates were incubated for 1 hr at room temperature and washed generously. Then, 50 μl of Streptavidin HRP-conjugated detection antibody (Invitrogen) diluted 1:10,000 in dilution buffer was added to each well, followed by a 1-hr incubation at room temperature. Plates were then thoroughly washed before being developed with 50 μl of 3,3',5,5'-Tetramethylbenzidine (EMD Millipore) at room temperature. This reaction was stopped with 50 μl of TMB Stop Solution (Southern Biotech) after a brief incubation. Endpoint titer was determined to be the highest dilution at which the optical density of the post-treatment plasma was greater than two times that of the pre-treatment plasma.

### SIV neutralization assays

Sera from the research animals were screened for neutralization of SIVmac239 and SIVmac316 utilizing the luciferase-based, TZM-bl assay, as described previously [54]. Stocks of replication-competent SIVmac239 and SIVmac316 were produced by transfecting HEK293T cells (ATCC) with full-length DNA using the jetPRIME technology (Polyplus transfection). Supernatant was harvested after 72 hrs and stored at −80°C until use. Neutralization was tested by incubating SIVmac239 or SIVmac316 and monkey sera for 1 hr at 37°C before transferring them onto TZM-bl cells (AIDS Research and Reference Reagent Program, Division of AIDS, NIAID, NIH). Neutralization was measured in duplicate wells within each experiment. Neutralization was tested starting at 1:4 serum dilutions followed by eight serial 2-fold dilutions for SIVmac239 or eight 4-fold serial dilutions for SIVmac316. The ID_50_ titer was defined by the Sigmoidal, 4PL, X is log(concentration) equation in Prism7 (GraphPad Software).

### Antibody-dependent cellular cytotoxicity (ADCC) assay

The SIVmac239Δ*vif* and SHIVAD8-EOΔ*vif* stocks used in the ADCC assays were produced by transfection of infectious molecular clones into HEK293T cells using GenJet transfection reagent (SignaGen). Virus-containing supernatants were collected 48 and 72 hr post-transfection and stored at −80°C. The original SHIVAD8-EO clone was provided by Dr. Malcom Martin (NIAID, Bethesda, MD). After heat inactivation for 30 min at 56°C, RM plasma samples were tested for non-specific ADCC due to the presence of antibodies to human cellular antigens by co-incubating uninfected CEM.NKR-CCR5-sLTR-Luc target cells (AIDS Research and Reference Reagent Program, Division of AIDS, NIAID, NIH) with an NK cell line (KHYG-1 cells) expressing rhesus CD16 at a 10:1 effector-to-target ratio in the presence of serial dilutions of plasma [55]. This NK cell line was developed in house, as described previously [55]. Non-specific lysis was detected as a reduction in background luciferase activity (% RLU) for target cells incubated with NK cells in the presence compared to the absence of plasma. Plasma samples that directed ADCC against uninfected cells were depleted of anti-human Abs by repeated cycles of incubation with CEM.NKR-CCR5-sLTR-Luc cells, followed by centrifugation and plasma transfer, until ADCC responses to uninfected cells were no longer detectable.

To measure ADCC activity in plasma of vaccinated animals, CEM.NKR-CCR5-sLTR-Luc target cells were infected with SIVmac239Δ*vif* or SHIVAD8-EOΔ*vif* (internal negative control) by spinoculation for 3 hr at 1200 × *g* in the presence of 40 μg/ml polybrene (EMD Millipore). Four days after infection, target cells were incubated with the NK cell line KHYG-1 at a 10:1 effector-to-target ratio in the presence of serial plasma dilutions. Luciferase activity was measured after 8 hr using the britelite plus luciferase assay system (PerkinElmer). Triplicate wells were tested at each plasma dilution, and wells containing effector cells incubated with uninfected or infected target cells in the absence of plasma were used to determine background and maximal luciferase activity, respectively. Changes in RLUs under each plasma dilution were used to calculate AUC values for SIVmac239- and SHIV-AD8-infected cells. The area under the SHIV-AD8 curve was subtracted from the area under the SIVmac239 curve, thereby yielding the rAUC value for each animal. These rAUC values were used as a measure of ADCC activity.

### Quantification of Mamu-A*01 tetramer+ CD8+ T-cells in PBMC

Mamu-A*01 tetramers bound to peptides corresponding to the Tat_28-35_SL8, Env_233-241_CL9, Env_620-628_TL9, and Vif_100-109_VL10 epitopes were obtained from the NIH Tetramer Core Facility. These tetramers were labelled with phycoerythrin (PE), allophycocyanin (APC), or Brilliant Violet (BV)421. The Mamu-A*01/Gag_181-189_CM9 tetramer was obtained from MBL International. These reagents were used to quantify SIV-specific CD8+ T-cells in PBMC, as described previously [56]. For the time-course analysis of vaccine-induced SIV epitope-specific CD8+ T-cell responses in Groups 1a and 2a, approximately 800,000 PBMC were incubated in R10 medium with titrated amounts of each tetramer at room temperature for 45 min. The cells were then stained with fluorochrome-labeled mAbs directed against the surface molecules CD3 (clone SP34-2; PerCP Cy5.5), CD8α (clone RPA-T8; BV785), CD14 (clone M5E2; BV510), CD16 (clone 3G8; BV510), and CD20 (clone 2H7; BV510) for 25 min at room temperature. This surface staining mAb cocktail also included an amine-reactive dye (ARD; Live/DEAD Fixable Aqua Dead Cell Stain; Life Technologies). The cells were then washed with Wash Buffer (Dulbecco's PBS with 0.1% bovine serum albumin and 0.45 g/L NaN_3_) and fixed with 1× BD FACS Lysing Solution (BD Biosciences) for 10 min at room temperature. The cells were washed one more time before they were acquired on a BD LSR II cytometer equipped with a 50-mW 405-nm violet laser, a 100-mW 488-nm blue laser, and a 30-mW 635-nm red laser using the FACSDIVA (version 6) software.

To determine the memory phenotype of vaccine-induced SIV-specific CD8+ T-cells in Groups 1a and 2a at the time of the first SIV challenge, 2.4×10^6^ PBMCs were incubated with an APC-conjugated Mamu-A*01/Gag_181-189_CM9 tetramer at room temperature for 45 min. The cells were then stained with the same mAbs specific for CD8α, CD14, CD16, and CD20 described above, plus mAbs against CD28 (clone 28.2; PE Cy7) and CCR7 (clone 150503; FITC). ARD Aqua was also included in this surface staining mAb cocktail. After a 25-min incubation at room temperature, cells were treated with 1× BD FACS Lysing Solution (BD Biosciences) for 10 min and subsequently washed with “Wash Buffer” (Dulbecco’s PBS with 0.1% BSA and 0.45 g/L NaN_3_). Cells were then permeabilized by treatment with “Perm buffer” [1X BD FACS Lysing Solution 2 (Beckton Dickinson) and 0.05% of Tween-20 (Sigma-Aldrich)] for 10 min, washed, and stained with a mAb against CD3 (clone SP34-2). After a 30-min incubation in the dark at room temperature, cells were washed and stored at 4°C until acquisition. Samples were acquired in the same flow cytometer described above.

FlowJo 9.9 (FlowJo, LLC) was used to analyze the data. Doublets were first excluded by gating out events with disproportionally high width in a forward scatter (FS) area vs. FS width plot, and then in a side scatter (SC) area vs. SC width plot. Next, a time gate was created that included only those events that were recorded within the 15^th^ and 85^th^ percentiles of acquisition time. The resulting cells were then gated on "dump channel" (CD14, CD16, CD20, ARD) negative, CD3+ cells. Because MHC-I tetramer binding to the T-cell receptor (TCR) can lead to TCR internalization, the CD3 gate at this stage included cells that expressed intermediate levels of CD3. Next, the lymphocyte population was delineated based on its FS and SS properties and subsequent analyses were conducted within CD8+ cells. After outlining MHC-I tetramer+ cells, the memory phenotyping analysis was performed within this gate. Rhesus macaque memory T-cells can be classified into three subsets based on the differential expression of CD28 and CCR7: central memory (T_CM_; CD28+CCR7+), transitional memory (T_EM1_; CD28+CCR7−), and fully-differentiated effector memory (T_EM2_; CD28−CCR7−). Cells stained with fluorochrome-labeled mAbs of the same isotypes as the anti-CD28 and anti-CCR7 mAbs guided the identification of the memory subsets within the MHC-I tetramer+ population. Based on this gating strategy, all tetramer frequencies mentioned in this manuscript correspond to percentages of live CD14− CD16− CD20− CD3+ CD8+ tetramer+ lymphocytes (S10 Fig).

### Intracellular cytokine staining (ICS) assay

The antigen stimuli for the ICS assays consisted of 16 pools of SIVmac239 15mer peptides overlapping by 11 amino acids corresponding to Gag amino acids 1–263 and 253–510; Pol amino acids 1–354, 344–700, and 690–1060; and Env amino acids 1–175, 161–355, 340–531, 516–707, and 692–879. Individual pools were used containing peptides that covered the entire ORFs of Vif, Vpx, Vpr, Tat, Rev, and Nef. The final concentration of each 15mer in the ICS tubes was 0.1–1.0 μM, depending on the peptide pool. Leukocyte Activation Cocktail (LAC; BD Pharmingen) and tissue culture medium devoid of stimulatory peptides were used as the positive and negative controls, respectively. The assay was set up in 12 × 75-mm polypropylene tubes (Corning, Inc.), each containing 1.6×10^6^ freshly isolated PBMC in a final volume of 1.0 ml of R10. The medium also contained unlabeled co-stimulatory mAbs against CD28 and CD49d, and a PE-conjugated mAb specific for CD107a. After adding the appropriate stimulation cocktails to each tube, the cells were placed in a 5.0% CO_2_ incubator at 37°C for 9 hr and then refrigerated to 4°C until staining with fluorochrome-labeled mAbs. To inhibit protein transport, Brefeldin A (Biolegend, Inc.) and GolgiStop (BD Biosciences) were added to all tubes 1 hr into this incubation period at concentrations of 5.0 μg/ml and 0.7 μg/ml, respectively. The surface staining master mix included mAbs against CD4 (clone OKT4; BV605) and CD8 (clone RPA-T8; BV785), in addition to the same BV510-conjugated mAbs against CD14, CD16, and CD20, and the ARD AQUA reagent described above. For the ICS step, the cells were fixed with BD FACS Lyse buffer and subsequently permeabilized with Perm Buffer, as described above. The cells were then incubated for 1 hr in the dark at room temperature with mAbs against CD3 (clone SP34-2; PerCP Cy5.5), IFN-γ (clone 4S.B3; BV421), TNF-α (clone Mab11; APC), and CD69 (clone FN50; PE Cy7). Once this incubation was completed, the cells were washed and stored at 4°C until acquisition in the same flow cytometer described above.

FlowJo 9.9 (FlowJo, LLC) was used to analyze the data. The same gating strategy described above was used to exclude doublets, and gate on live CD14− CD16− CD20− CD3+ lymphocytes acquired within the 15^th^ and 85^th^ percentiles of time. Next, T-cell subsets were analyzed based on their expression of either CD4 or CD8, but not both markers. Functional analyses were conducted within these two compartments by creating gates for each function (IFN-γ, TNF-α, and CD107a). The Boolean gate platform was used to generate a full array of possible combinations, equating to 8 response patterns when testing 3 functions (2^3^ = 8). Cells were considered positive for IFN-γ, TNF-α, or CD107a if these molecules were co-expressed with CD69, a marker of recent activation. At time points of robust SIV-specific CD8+ T-cell activation, a fraction of the CD8+ T-cells that expressed high levels of the degranulation marker CD107a exhibited low to intermediate levels of CD69. In those cases, all CD107a-expressing cells were considered to be positive based on the lack of background CD107a staining in the negative control tests. LAC-stimulated cells stained with fluorochrome-labeled control mAbs of the same isotypes as those against IFN-γ, TNF-α, and CD107a guided the identification of positive populations. Two criteria were used to determine whether a response was positive. First, the frequency of gated events had to be ≥2-fold higher than their corresponding values in background-subtracted negative control tests. Second, the gates for each response had to contain ≥10 events. These calculations were performed with Microsoft Excel and results were presented as the percentages of responding CD4+ or CD8+ T-cells, that is, live CD14− CD16− CD20− CD3+ lymphocytes of either subset producing any combination of IFN-γ, TNF-α, or CD107a (S11 Fig).

### Quantification of activated CD4+ T-cell subsets in PBMC

Cryopreserved PBMC samples collected at study week 50 (i.e., 3–4 weeks prior to the first SIV challenge) were used in this assay. First, cell suspensions from each animal were stained with LIVE/DEAD Fixable Aqua Dead Cell Stain Kit (Life Technologies) for 30 min at room temperature. Next, a cocktail of fluorochrome-labeled mAbs directed against CD3 (PerCP Cy5.5), CD4 (BV605), CD8α (BV785), CD14 (BV510), CD16 (BV510), CD20 (BV510), CCR5 (clone 3A9; PE), and HLA-DR (clone G46-6; APC) was added to the cells. Except for the latter two mAbs, the clones for all mAbs were the same as the ones described above for the tetramer staining and ICS assays. After a 30-min incubation at room temperature, the cells were fixed and permeabilized utilizing the Transcription Factor Buffer Set kit (BD Biosciences) according to the manufacturer’s instructions. The intracellular staining step included a mAb against ki-67 (clone B56; BV421). Data was collected using the BD LSR II flow cytometer described above. FlowJo 10.5.3 (FlowJo, LLC) was used to analyze the data. The same gating strategy described above was used to exclude doublets, and gate on live CD14− CD16− CD20− CD3+ lymphocytes acquired within the 15^th^ and 85^th^ percentiles of time. Because CD4+ T-cells showed little or no staining with the anti-HLA-DR mAb, HLA-DR expression was not considered for this analysis. The percentages of cells expressing ki-67 and/or CCR5 were determined within a CD4+ CD8− gate.

### Plasmablast IgG enzyme-linked immune absorbed spot (ELISpot) assay

ELISpot assays were performed based on a previous protocol [57]. The assays were carried out according to the manufacturer’s instructions (Mabtech, Inc.) and used 120,000 purified plasmablasts per well. To measure total IgG-secreting cells and SIV Env-specific IgG-secreting cells, the plates were coated with either an anti-human IgG mAb (Mabtech, Inc., 15.0 μg/ml) or purified SIVmac239 gp140 (Immune-Tech, 1.0 μg/ml), respectively. RM plasmablasts were sorted from freshly isolated PBMC utilizing a FACSJazz (BD Biosciences, San Jose, CA) cell sorter equipped with a 488-nm blue laser and a 640-nm red laser. The cells were sorted based on the following phenotype: lymphocytes / singlets / live / CD3− CD16− CD20(neg/int) / HLA-DR+ / CD14− CD11c− CD123− / CD80+. Wells were imaged and spots were enumerated with an AID ELISpot reader (AID). Assay results are shown as spot-forming cells (SFC) per 10^6^ cells. To obtain positive and negative controls for this assay, SIV-infected and SIV naïve RMs were bled 4–7 days prior to each ELISpot assay and their PBMCs were cultured in R10 in the presence of R-848 (1.0 μg/ml) and rhesus interleukin-2 (10.0 ng/ml). On the day of the ELISpot assays, these cultures were washed extensively and then subjected to the same cell sorting procedure as the freshly isolated PBMC from the Group 1 and Group 2 animals.

### Statistics

To determine whether the rate of SIVmac239 acquisition differed between Groups 1 and 3, and between Groups 2 and 3, the time to productive infection was analyzed using the Cox proportional hazard model. Groups 1 and 3, and Groups 2 and 3, were also compared in terms of the number of infected versus uninfected animals after six SIV challenges using Fisher’s exact test. These same comparisons were performed using an expanded control group consisting of the eight contemporaneous monkeys in Group 3 plus 52 historical control animals. For comparing immunological variables between Groups 1 and 2, median regression was used when the variable was expressed as a proportion or ratio [58]. In all other cases, Welch’s t-test was used for these comparisons. Welch’s t-test was also used for comparing peak and setpoint viral loads between Groups 1–3. To quantitatively assess the association between infection rate and pre-challenge attributes of vaccine-induced SIV-specific responses, we performed Cox proportional hazard regression using the number of SIV challenges as the outcome and immunological responses as primary predictors. As the scale of immunological responses varies, we also performed the same analysis using standardized values (also known as Z-score), which results in different hazard ratios (reported in Table 1) but identical P values as the classical analysis. We searched for associations between pre-challenge attributes of vaccine-induced SIV-specific responses and control of viral replication in SIV-infected vaccinees using the Spearman rank correlation method. To test the difference in immunological responses over time, we performed mixed effect median regression, using time and group-by-time interaction as fixed effects, and individual difference as a random effect. The type I error rate for all the tests was fixed at 0.05 (2-tailed).

## Supporting information

S1 Fig Scheme of SIV vaccine inserts. Seven different vaccine vectors were used in this experiment. A-B) Vectors 1 and 2 were based on the pCMVkan backbone, which utilizes the CMV enhancer/promoter to drive transgene expression. Vectors 1 and 2 contained SIVnfl inserts that differed in the Env proteins they expressed. The SIVnfl insert in vector 1 expressed a truncated version of SIVmac239 Env (E_767_Stop) intended to increase Env surface expression (A), while the SIVnfl insert present in vector 2 expressed an intact SIVmac316 Env protein (B). Both vectors 1 and 2 contained a 6-base pair (bp) deletion in *nef* (nucleotides 9,791–9,796), corresponding to amino acids 239–240, that abrogates Nef-mediated major histocompatibility complex class-I (MHC-I) down-regulation. Additionally, the thymine at position 6,405 of the *tat* gene in vectors 1 and 2 was mutated to adenine to cause an L_35_Q substitution. This mutation was intended to prevent the immunodominant Mamu-A*01-restricted Tat_28-35_SL8 epitope from binding to the Mamu-A*01 molecule [22]. C-G) Vectors 3–7 were based on the RRV 26–95 backbone described previously [29]. Three of these constructs contained the SIVnfl insert, albeit under the control of different promoters. Vector 3 contained the CMV enhancer/promoter placed upstream of the SIVnfl insert. In vector 4, a hybrid early/late promoter construct consisting of the late promoter for RRV ORF26 (p26) and the early promoter for the RRV Poly Adenylated Nuclear RNA (PAN) was inserted just upstream of the SIVnfl insert. In vector 5, the SIV promoter/enhancer region was used by restoring nucleotides 1–521 of the 5’ LTR. These three promoters were used in combination in an attempt to achieve stable expression of SIVnfl during all stages of the RRV life cycle. In order to maximize *in vivo* expression of SIV Env, two additional rRRV constructs encoded SIV *env* alone under the control of the p26 promoter. Vector 6 encoded SIVmac239 *env* and vector 7 encoded the closely related SIVmac316 *env*. The codon usage of these *env* inserts was modified to reflect the codon usage of RRV glycoprotein in order to allow adequate expression in monkeys [52]. Both SIVmac239 and SIVmac316 *env* inserts were preceded by the splicing donor sequence (SD, AAACAAGTAAGT) and contained the aforementioned E_767_Stop truncation. Promoters are indicated by gray boxes. Bovine growth hormone polyadenylation (BGH polyA) signals are indicated by green boxes. The *nef* open reading frame in the SIVnfl inserts in vectors 3–5 contained a C-terminal V5 tag, which is indicated by cyan boxes. The SIVmac316 *env* sequences in vectors 2 and 7 are indicated by orange boxes. All other sequences are of SIVmac239 origin. Nucleotide and amino acid numberings are based on the SIVmac239 genome.
(PDF)

S2 Fig Kinetics of vaccine-induced CD8+ T-cell responses targeting Mamu-A*01-restricted SIV epitopes. Fluorochrome-labeled Mamu-A*01 tetramers folded with peptides corresponding to SIV epitopes were used to track vaccine-elicited CD8+ T-cells in PBMC from the Group 1a (left column) and Group 2a (right column). The percentages of live tetramer+ CD8+ T-cells specific for Vif_100-109_VL10 (A), Env_620-628_TL9 (B), Env_233-241_CL9 (C), and Tat_28-35_SL8 (D) are shown at multiple time points throughout the vaccine phase. The time scale in the x-axes matches that in Fig 1.
(PDF)

S3 Fig Kinetics of peripheral blood lymphocyte subsets during the rDNA-SIVnfl priming phase. Flow cytometric analysis of PBMC and contemporaneous white blood cell counts were used to determine the absolute numbers of lymphocyte subsets during the rDNA-SIVnfl priming immunizations of the Group 1b (left column) and Group 2b (middle column) monkeys. Based on these numbers, the fold-change from baseline was calculated for each animal and plotted against time. A) Total T-cell counts (live CD14− CD16− CD20− CD3+ lymphocytes). B) CD4+ T-cell counts (live CD14− CD16− CD20− CD3+ CD4+ CD8− lymphocytes). C) CD8+ T-cells (live CD14− CD16− CD20− CD3+ CD4− CD8+ lymphocytes). D) T regulatory cells (Tregs; live CD14− CD16− CD20− CD3+ CD4+ CD8− CD25+ FoxP3+ lymphocytes). E) B-cells (live CD14− CD16− CD20+ lymphocytes). The panels on the right show group means for each lymphocyte subset. The error bars in the right panels correspond to the standard error of the mean and each symbol in the left and middle panels denotes one vaccinee. Differences in the levels of each lymphocyte subset between Groups 1b and 2b were evaluated using mixed-effect median regression, using time and group-by-time interactions as fixed effects, and individual differences as random effects. Time points when statistically significant differences between Groups 1b and 2b were found are indicated by asterisks on the panels on the right.
(PDF)

S4 Fig Kinetics of vaccine-induced CD4+ T-cell responses against Gag, Env, and Nef. ICS was used to quantify vaccine-induced CD4+ T-cell responses against Gag (A), Env (B), and Nef (C) in Groups 1b (left column) and 2b (middle column) at multiple time points during the vaccine phase. Group means for these responses are shown in the right column. The error bars in the right panels correspond to the standard error of the mean and each symbol in the left and middle panels denotes one vaccinee. The time scale in the x-axes matches that in Fig 1. The percentages of responding CD4+ T cells shown in the y-axes were calculated by adding the background-subtracted frequencies of positive responses producing any combination of IFN-γ, TNF-α, and CD107a. To search for differences in vaccine-induced CD4+ T-cell responses over time between Groups 1b and 2b, mixed-effect quantile regression was performed, using time and group-by-time interactions as fixed effects, and individual differences as random effects. Time points when statistically significant differences between Groups 1b and 2b were found are indicated by asterisks on the panels on the right.
(PDF)

S5 Fig Kinetics of vaccine-induced CD4+ T-cell responses against Pol, Vif, Vpx, Vpr, Tat, and Rev. ICS was used to quantify vaccine-induced CD4+ T-cell responses against Pol (A), Vif (B), Vpx (C), Vpr (D), Tat (E), and Rev (F) in Groups 1b (left column) and 2b (middle column) at multiple time points during the vaccine phase. Group means for these responses are shown in the right column. Error bars correspond to the standard error of the mean and each symbol denotes one vaccinee. The time scale in the x-axes matches that in Fig 1. The percentages of responding CD4+ T cells shown in the y-axes were calculated by adding the background-subtracted frequencies of positive responses producing any combination of IFN-γ, TNF-α, and CD107a. To search for differences in vaccine-induced CD4+ T-cell responses over time between Groups 1b and 2b, mixed-effect quantile regression was performed, using time and group-by-time interactions as fixed effects, and individual differences as random effects. Significant group-by-time interactions were not observed.
(PDF)

S6 Fig Lack of sex differences in vaccine-induced SIV-specific immune responses. Vaccine-induced SIV-specific immune responses measured at the time of the 1^st^ SIV challenge were compared between males and females in Group 1 (1a+1b) and Group 2 (2a+2b) in terms of CD8+ T-cell breadth (A) and magnitude (B); midpoint titers of gp140-binding (C) and gp120-binding (D) antibodies (Abs); the lowest reciprocal dilution that results in 50% reduction of SIVmac316 infectivity in TZM-bl assays (ID_50_; E); and NK cell-mediated Ab-dependent cellular cytotoxicity (ADCC) activity against SIVmac239-infected cells (F). As a reference, the mean ID_50_ titer of animals infected with SIVmac239Δ*nef* for 23 weeks is shown in E as horizontal dashed lines. The ADCC activity was calculated as described in the Materials and Methods section. As a reference, the rAUC value for pooled plasma from SIVmac239-infected RMs is shown in F as horizontal dotted lines. Lines correspond to mean values and each symbol denotes one vaccinee. *P*-values were calculated using Welch’s t-test.
(PDF)

S7 Fig Quantification of Env-specific B-cells in PBMC induced by the rDNA-SIVnfl (Group 1) and rDNA-SIVnfl+Ipi (Group 2) priming immunizations. Plasmablasts were sorted from PBMC from each animal in Group 1 (1a+1b) and Group 2 (2a+2b) on the day of the 4^th^ rDNA-SIVnfl prime and seven days later. These cells were then used in IgG ELISPOT assays for quantification of SIVmac239 gp140-specific plasmablasts. A-B) The frequency of gp140-specific plasmablasts on days 0 and 7 post 4^th^ rDNA-SIVnfl prime is shown for Groups 1b and 2b (A), and for Groups 1a and 2a (B). C-D) The frequencies of gp140-specific plasmablasts measured on day 7 post 4^th^ rDNA-SIVnfl prime were compared between Groups 1b and 2b (C), and between Groups 1a and 2a (D). Results are shown as spot-forming cells (SFC) per 10^6^ plasmablasts. Lines correspond to mean values and each symbol denotes one vaccinee. *P*-values were calculated using Welch’s t-test.
(PDF)

S8 Fig Levels of vaccine-induced gp140-binding IgG antibodies in rectal secretions from the Group 1 and Group 2 animals. Weck-cel sponges were used to collect rectal secretions from each of the monkeys in Group 1 (1a+1b) and Group 2 (2a+2b) at study week 48. IgG reactivity to SIVmac239 gp140 and the total IgG concentrations were determined by ELISA. The ratio of gp140-specific IgG/total IgG was used to compare the levels of vaccine-induced gp140-binding IgG antibodies in rectal fluid between Groups 1 and 2. Lines correspond to mean values and each symbol denotes one vaccinee. *P*-values were calculated using median regression, with Group 2 as the reference group.
(PDF)

S9 Fig Frequencies of vaccine-induced SIV-specific T-cell responses in protected vaccinees before and after the SIV challenge phase. A-B) Three vaccinees in Group 1b and two in Group 2b resisted 6 IR challenges with SIVmac239. PBMCs from these animals were obtained 2.3 weeks after the 6^th^ SIV exposure and used in ICS assays containing peptides covering the entire SIVmac239 proteome. The total magnitude of vaccine-induced SIV-specific CD8+ (A) and CD4+ (B) T-cell responses measured at this timepoint was compared to that measured at the week of the 1^st^ SIV challenge. The percentages of responding CD8+ T cells shown in the *y*-axes were calculated by adding the background-subtracted frequencies of positive responses producing any combination of IFN-γ, TNF-α, and CD107a. C) Two vaccinees in Group 1a and one in Group 2a resisted 6 IR challenges with SIVmac239. PBMCs from these animals were obtained 2.3 weeks after the 6^th^ SIV exposure and stained with a fluorochrome-labeled Mamu-A*01/Gag_181-189_CM9 tetramer. The frequency of tetramer+ CD8+ T-cells measured at this time point was compared to that measured at the week of the 1^st^ SIV challenge. Each symbol denotes one vaccinee. *P*-values were calculated using Welch’s t-test.
(PDF)

S10 Fig Gating strategy for the analysis of MHC-I+ CD8+ T-cells. (PDF)

S11 Fig Gating strategy for the ICS analysis. (PDF)
