# Supplementary material for: Vaccine protection against rectal acquisition of SIVmac239 in rhesus macaques
Source: PLoS Pathog. 2019 Sep 30;15(9):e1008015. doi: 10.1371/journal.ppat.1008015 (PMC6791558; doi:10.1371/journal.ppat.1008015)
Supplement: S1 Fig — Seven different vaccine vectors were used in this experiment. A-B) Vectors 1 and 2 were based on the pCMVkan backbone, which utilizes the CMV enhancer/promoter to drive transgene expression. Vectors 1 and 2 contained SIVnfl inserts that differed in the Env proteins they expressed. The SIVnfl insert in vector 1 expressed a truncated version of SIVmac239 Env (E767Stop) intended to increase Env surface expression (A), while the SIVnfl insert present in vector 2 expressed an intact SIVmac316 Env protein (B). Both vectors 1 and 2 contained a 6-base pair (bp) deletion in nef (nucleotides 9,791–9,796), corresponding to amino acids 239–240, that abrogates Nef-mediated major histocompatibility complex class-I (MHC-I) down-regulation. Additionally, the thymine at position 6,405 of the tat gene in vectors 1 and 2 was mutated to adenine to cause an L35Q substitution. This mutation was intended to prevent the immunodominant Mamu-A*01-restricted Tat28-35SL8 epitope from binding to the Mamu-A*01 molecule [22]. C-G) Vectors 3–7 were based on the RRV 26–95 backbone described previously [29]. Three of these constructs contained the SIVnfl insert, albeit under the control of different promoters. Vector 3 contained the CMV enhancer/promoter placed upstream of the SIVnfl insert. In vector 4, a hybrid early/late promoter construct consisting of the late promoter for RRV ORF26 (p26) and the early promoter for the RRV Poly Adenylated Nuclear RNA (PAN) was inserted just upstream of the SIVnfl insert. In vector 5, the SIV promoter/enhancer region was used by restoring nucleotides 1–521 of the 5’ LTR. These three promoters were used in combination in an attempt to achieve stable expression of SIVnfl during all stages of the RRV life cycle. In order to maximize in vivo expression of SIV Env, two additional rRRV constructs encoded SIV env alone under the control of the p26 promoter. Vector 6 encoded SIVmac239 env and vector 7 encoded the closely related SIVmac316 env. The codon usage of these e [file ppat.1008015.s001.pdf]

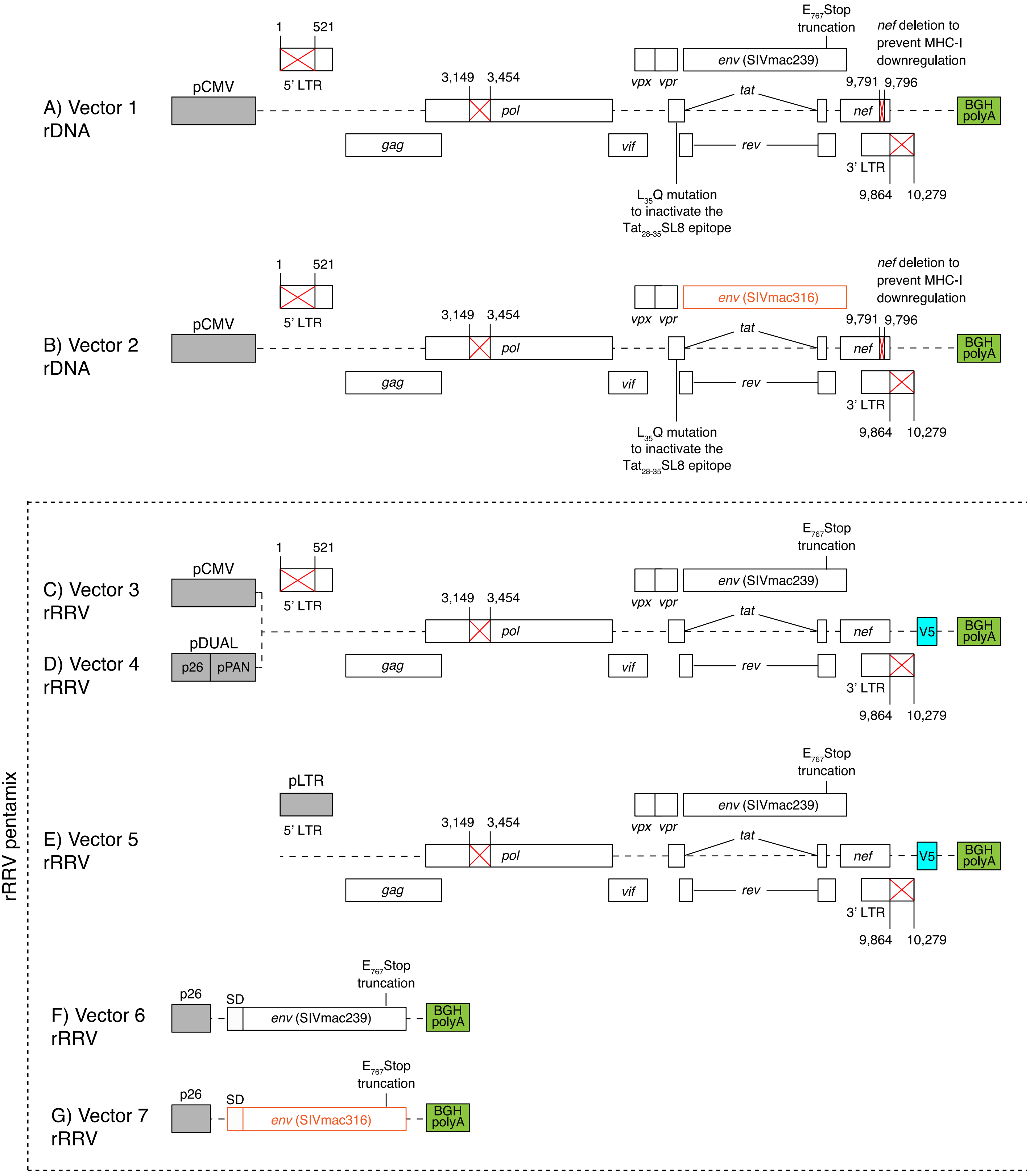
A) Vector 1 rDNA
pCMV
1
521
5' LTR
3,149
3,454
pol
gag
vif
vpx
vpr
env (SIVmac239)
E767Stop truncation
tat
rev
nef
nef deletion to prevent MHC-I downregulation
9,791
9,796
3' LTR
9,864
10,279
BGH polyA
L35Q mutation to inactivate the Tat28-35SL8 epitope
B) Vector 2 rDNA
env (SIVmac316)
C) Vector 3 rRRV
D) Vector 4 rRRV
pDUAL
p26
pPAN
V5
E) Vector 5 rRRV
pLTR
F) Vector 6 rRRV
SD
G) Vector 7 rRRV
rRRV pentamix
