# Supplementary material for: Vaccine protection against rectal acquisition of SIVmac239 in rhesus macaques
Source: PLoS Pathog. 2019 Sep 30;15(9):e1008015. doi: 10.1371/journal.ppat.1008015 (PMC6791558; doi:10.1371/journal.ppat.1008015)
Supplement: S2 Fig — Fluorochrome-labeled Mamu-A*01 tetramers folded with peptides corresponding to SIV epitopes were used to track vaccine-elicited CD8+ T-cells in PBMC from the Group 1a (left column) and Group 2a (right column). The percentages of live tetramer+ CD8+ T-cells specific for Vif100-109VL10 (A), Env620-628TL9 (B), Env233-241CL9 (C), and Tat28-35SL8 (D) are shown at multiple time points throughout the vaccine phase. The time scale in the x-axes matches that in Fig 1. (PDF) [file ppat.1008015.s002.pdf]

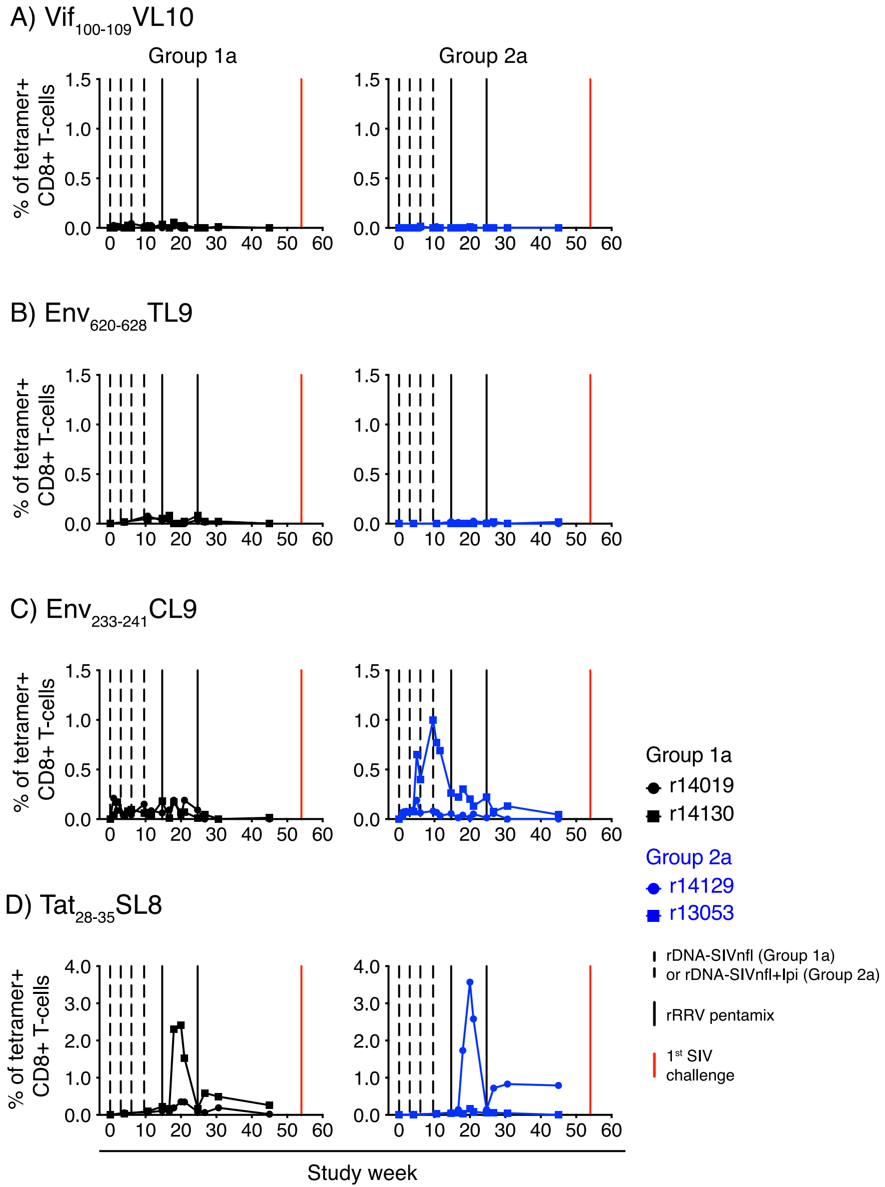
A) Vif100-109VL10
B) Env620-628TL9
C) Env233-241CL9
D) Tat28-35SL8
Group 1a
Group 2a
% of tetramer+ CD8+ T-cells
Study week
Group 1a
r14019
r14130
Group 2a
r14129
r13053
rDNA-SIVnfl (Group 1a)
or rDNA-SIVnfl+Ipi (Group 2a)
rRRV pentamix
1st SIV
challenge
