# Supplementary figures and images for: Vaccine protection against rectal acquisition of SIVmac239 in rhesus macaques
Source: PLoS Pathog. 2019 Sep 30;15(9):e1008015. doi: 10.1371/journal.ppat.1008015 (PMC6791558; doi:10.1371/journal.ppat.1008015)

A) T-cell counts

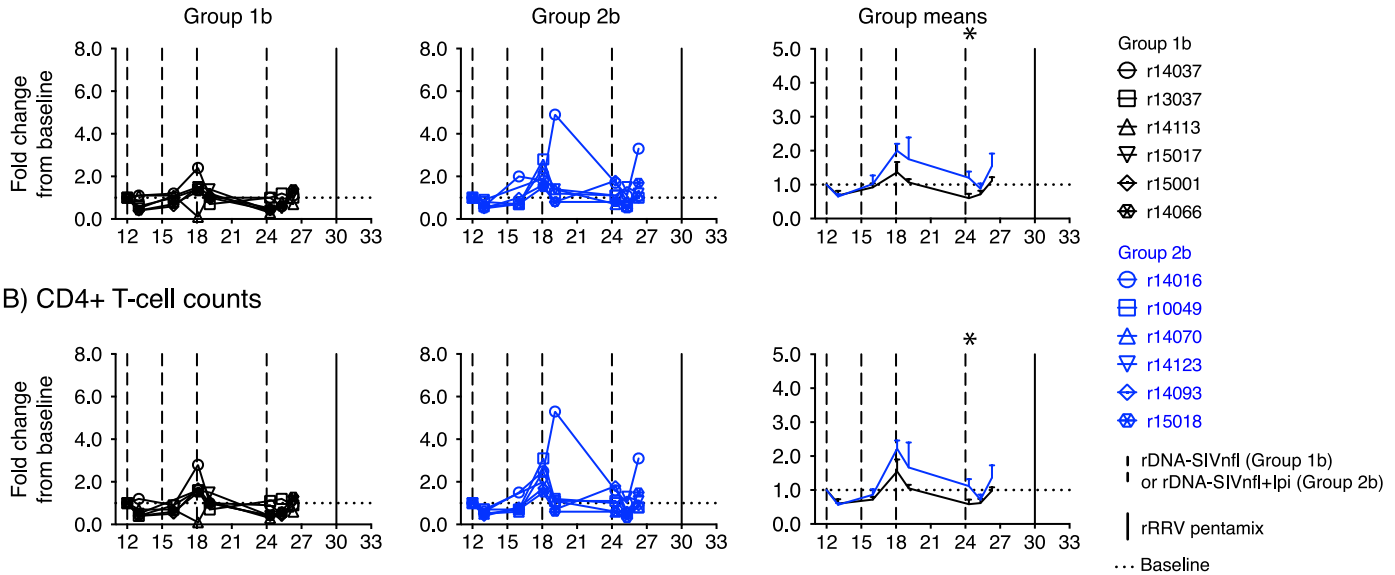

B) CD4+ T-cell counts

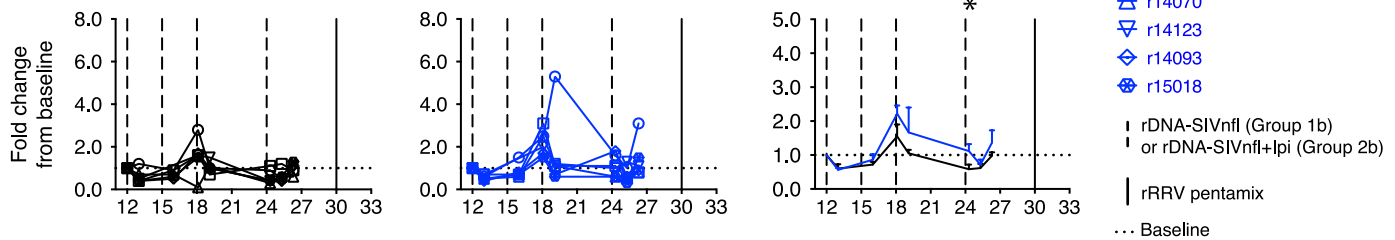

C) CD8+ T-cell counts

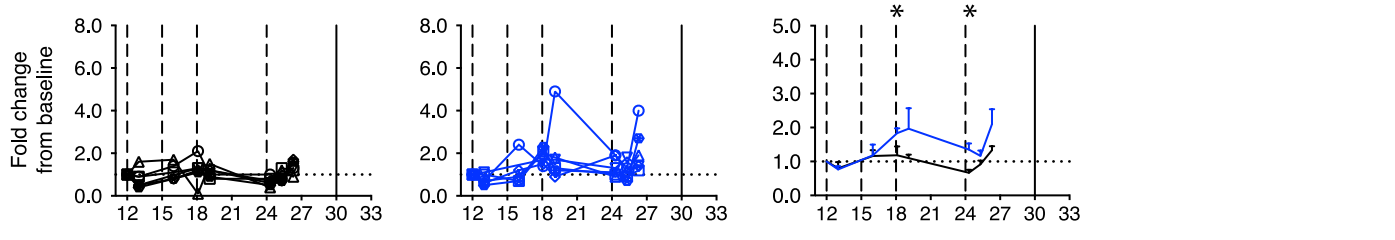

D) Treg cell counts

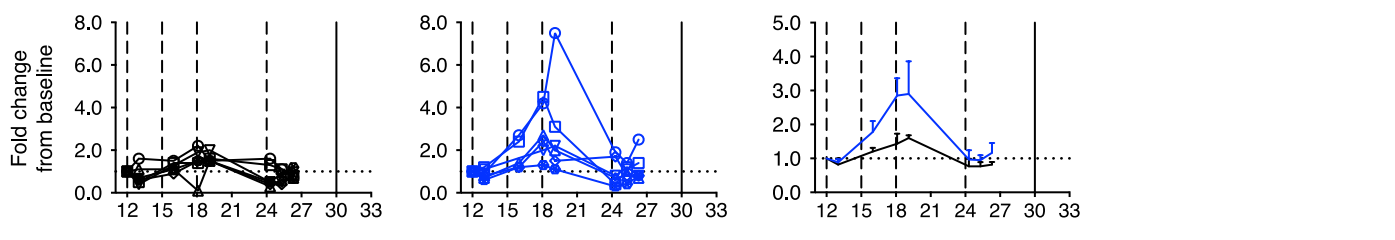

E) B-cell counts

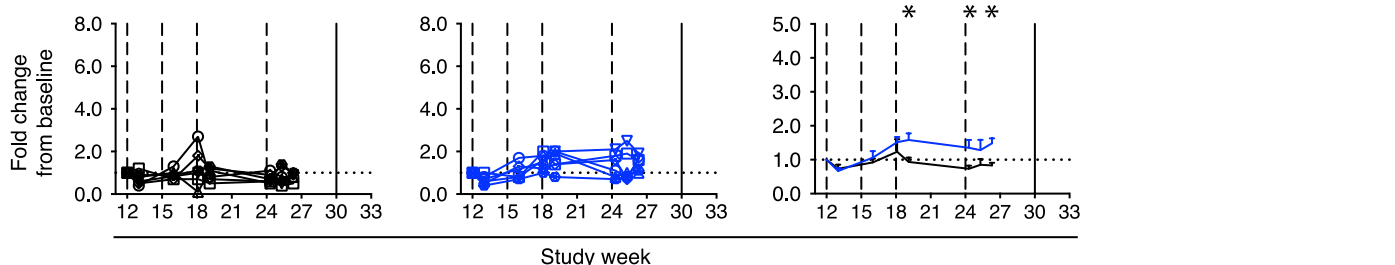

Supplement: S3 Fig — Flow cytometric analysis of PBMC and contemporaneous white blood cell counts were used to determine the absolute numbers of lymphocyte subsets during the rDNA-SIVnfl priming immunizations of the Group 1b (left column) and Group 2b (middle column) monkeys. Based on these numbers, the fold-change from baseline was calculated for each animal and plotted against time. A) Total T-cell counts (live CD14− CD16− CD20− CD3+ lymphocytes). B) CD4+ T-cell counts (live CD14− CD16− CD20− CD3+ CD4+ CD8− lymphocytes). C) CD8+ T-cells (live CD14− CD16− CD20− CD3+ CD4− CD8+ lymphocytes). D) T regulatory cells (Tregs; live CD14− CD16− CD20− CD3+ CD4+ CD8− CD25+ FoxP3+ lymphocytes). E) B-cells (live CD14− CD16− CD20+ lymphocytes). The panels on the right show group means for each lymphocyte subset. The error bars in the right panels correspond to the standard error of the mean and each symbol in the left and middle panels denotes one vaccinee. Differences in the levels of each lymphocyte subset between Groups 1b and 2b were evaluated using mixed-effect median regression, using time and group-by-time interactions as fixed effects, and individual differences as random effects. Time points when statistically significant differences between Groups 1b and 2b were found are indicated by asterisks on the panels on the right. (PDF) [file ppat.1008015.s003.pdf]

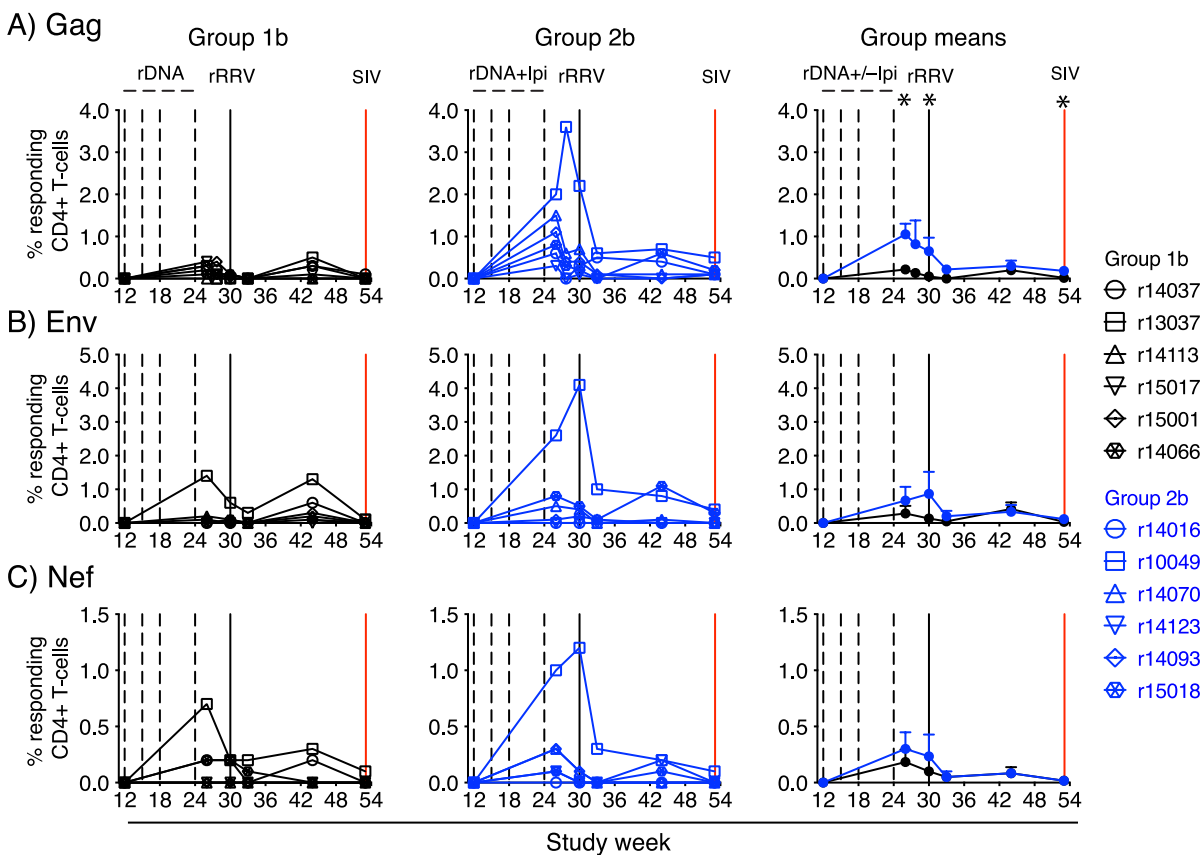

Supplement: S4 Fig — ICS was used to quantify vaccine-induced CD4+ T-cell responses against Gag (A), Env (B), and Nef (C) in Groups 1b (left column) and 2b (middle column) at multiple time points during the vaccine phase. Group means for these responses are shown in the right column. The error bars in the right panels correspond to the standard error of the mean and each symbol in the left and middle panels denotes one vaccinee. The time scale in the x-axes matches that in Fig 1. The percentages of responding CD4+ T cells shown in the y-axes were calculated by adding the background-subtracted frequencies of positive responses producing any combination of IFN-γ, TNF-α, and CD107a. To search for differences in vaccine-induced CD4+ T-cell responses over time between Groups 1b and 2b, mixed-effect quantile regression was performed, using time and group-by-time interactions as fixed effects, and individual differences as random effects. Time points when statistically significant differences between Groups 1b and 2b were found are indicated by asterisks on the panels on the right. (PDF) [file ppat.1008015.s004.pdf]

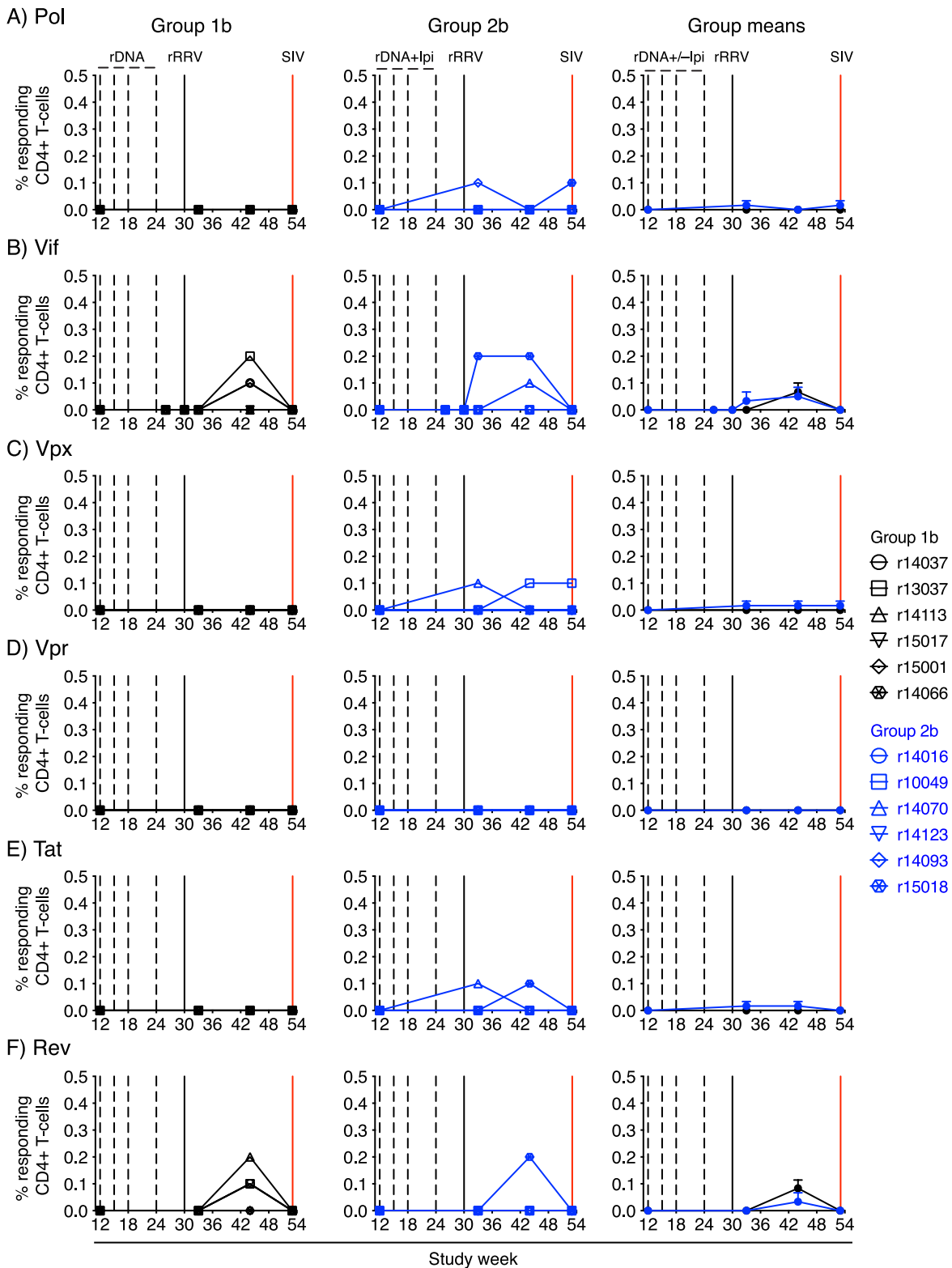

Supplement: S5 Fig — ICS was used to quantify vaccine-induced CD4+ T-cell responses against Pol (A), Vif (B), Vpx (C), Vpr (D), Tat (E), and Rev (F) in Groups 1b (left column) and 2b (middle column) at multiple time points during the vaccine phase. Group means for these responses are shown in the right column. Error bars correspond to the standard error of the mean and each symbol denotes one vaccinee. The time scale in the x-axes matches that in Fig 1. The percentages of responding CD4+ T cells shown in the y-axes were calculated by adding the background-subtracted frequencies of positive responses producing any combination of IFN-γ, TNF-α, and CD107a. To search for differences in vaccine-induced CD4+ T-cell responses over time between Groups 1b and 2b, mixed-effect quantile regression was performed, using time and group-by-time interactions as fixed effects, and individual differences as random effects. Significant group-by-time interactions were not observed. (PDF) [file ppat.1008015.s005.pdf]

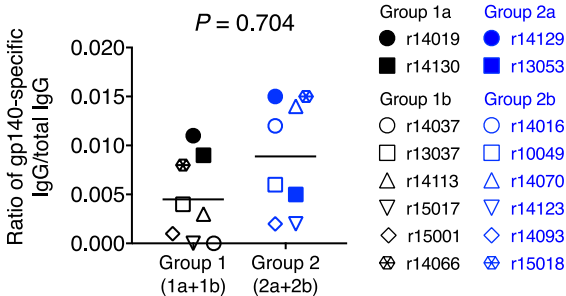

Supplement: S8 Fig — Weck-cel sponges were used to collect rectal secretions from each of the monkeys in Group 1 (1a+1b) and Group 2 (2a+2b) at study week 48. IgG reactivity to SIVmac239 gp140 and the total IgG concentrations were determined by ELISA. The ratio of gp140-specific IgG/total IgG was used to compare the levels of vaccine-induced gp140-binding IgG antibodies in rectal fluid between Groups 1 and 2. Lines correspond to mean values and each symbol denotes one vaccinee. P-values were calculated using median regression, with Group 2 as the reference group. (PDF) [file ppat.1008015.s008.pdf]
