# Supplementary material for: Vaccine protection against rectal acquisition of SIVmac239 in rhesus macaques
Source: PLoS Pathog. 2019 Sep 30;15(9):e1008015. doi: 10.1371/journal.ppat.1008015 (PMC6791558; doi:10.1371/journal.ppat.1008015)
Supplement: S6 Fig — Vaccine-induced SIV-specific immune responses measured at the time of the 1st SIV challenge were compared between males and females in Group 1 (1a+1b) and Group 2 (2a+2b) in terms of CD8+ T-cell breadth (A) and magnitude (B); midpoint titers of gp140-binding (C) and gp120-binding (D) antibodies (Abs); the lowest reciprocal dilution that results in 50% reduction of SIVmac316 infectivity in TZM-bl assays (ID50; E); and NK cell-mediated Ab-dependent cellular cytotoxicity (ADCC) activity against SIVmac239-infected cells (F). As a reference, the mean ID50 titer of animals infected with SIVmac239Δnef for 23 weeks is shown in E as horizontal dashed lines. The ADCC activity was calculated as described in the Materials and Methods section. As a reference, the rAUC value for pooled plasma from SIVmac239-infected RMs is shown in F as horizontal dotted lines. Lines correspond to mean values and each symbol denotes one vaccinee. P-values were calculated using Welch’s t-test. (PDF) [file ppat.1008015.s006.pdf]

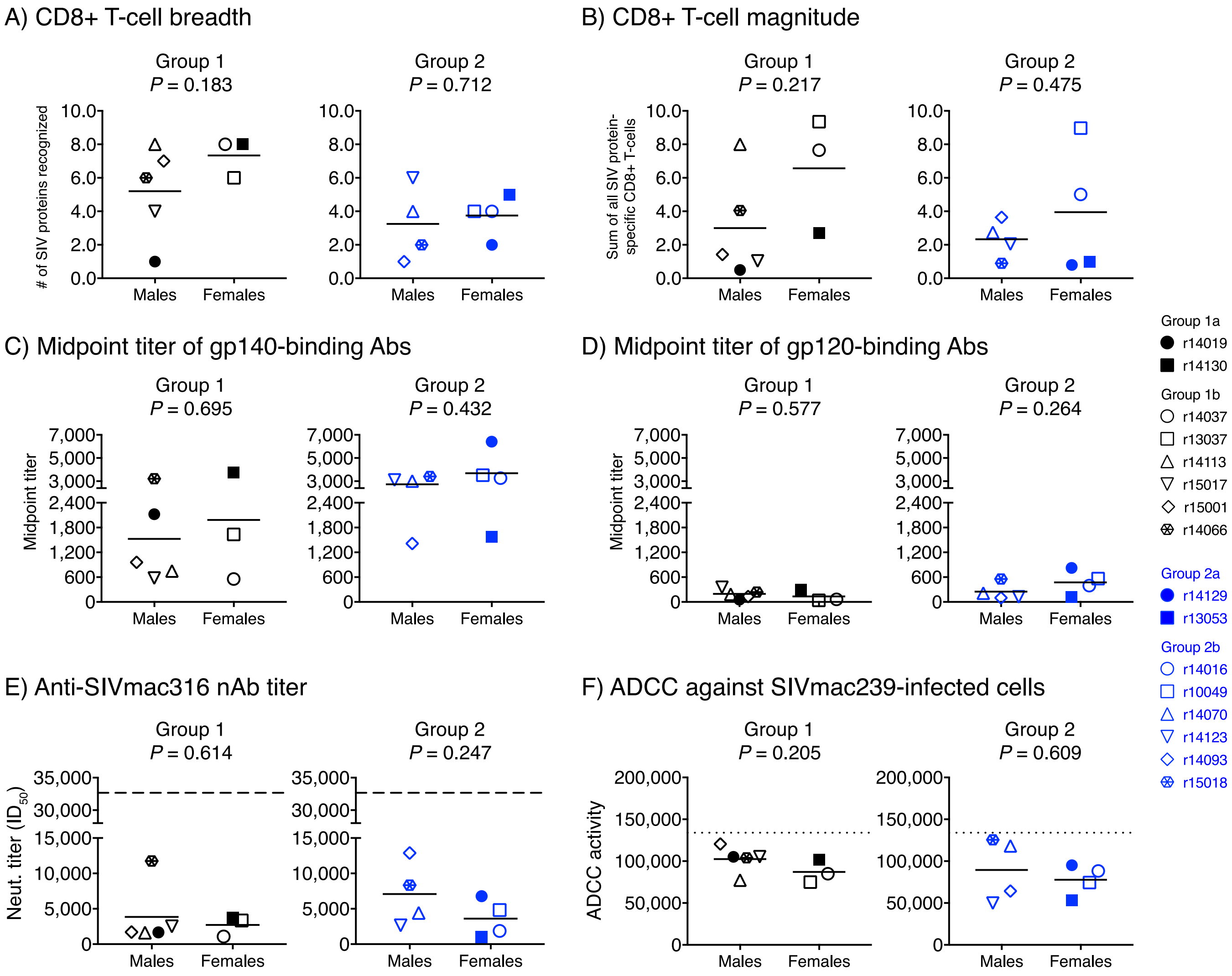
A) CD8+ T-cell breadth
Group 1
P = 0.183
Group 2
P = 0.712
# of SIV proteins recognized
10.0
8.0
6.0
4.0
2.0
0.0
Males
Females
B) CD8+ T-cell magnitude
Group 1
P = 0.217
Group 2
P = 0.475
Sum of all SIV protein-specific CD8+ T-cells
C) Midpoint titer of gp140-binding Abs
Group 1
P = 0.695
Group 2
P = 0.432
Midpoint titer
7,000
5,000
3,000
2,400
1,800
1,200
600
0
D) Midpoint titer of gp120-binding Abs
Group 1
P = 0.577
Group 2
P = 0.264
E) Anti-SIVmac316 nAb titer
Group 1
P = 0.614
Group 2
P = 0.247
Neut. titer (ID50)
35,000
30,000
15,000
10,000
5,000
0
F) ADCC against SIVmac239-infected cells
Group 1
P = 0.205
Group 2
P = 0.609
ADCC activity
200,000
150,000
100,000
50,000
0
Group 1a
r14019
r14130
Group 1b
r14037
r13037
r14113
r15017
r15001
r14066
Group 2a
r14129
r13053
Group 2b
r14016
r10049
r14070
r14123
r14093
r15018
