# Supplementary material for: Vaccine protection against rectal acquisition of SIVmac239 in rhesus macaques
Source: PLoS Pathog. 2019 Sep 30;15(9):e1008015. doi: 10.1371/journal.ppat.1008015 (PMC6791558; doi:10.1371/journal.ppat.1008015)
Supplement: S7 Fig — Plasmablasts were sorted from PBMC from each animal in Group 1 (1a+1b) and Group 2 (2a+2b) on the day of the 4th rDNA-SIVnfl prime and seven days later. These cells were then used in IgG ELISPOT assays for quantification of SIVmac239 gp140-specific plasmablasts. A-B) The frequency of gp140-specific plasmablasts on days 0 and 7 post 4th rDNA-SIVnfl prime is shown for Groups 1b and 2b (A), and for Groups 1a and 2a (B). C-D) The frequencies of gp140-specific plasmablasts measured on day 7 post 4th rDNA-SIVnfl prime were compared between Groups 1b and 2b (C), and between Groups 1a and 2a (D). Results are shown as spot-forming cells (SFC) per 106 plasmablasts. Lines correspond to mean values and each symbol denotes one vaccinee. P-values were calculated using Welch’s t-test. (PDF) [file ppat.1008015.s007.pdf]

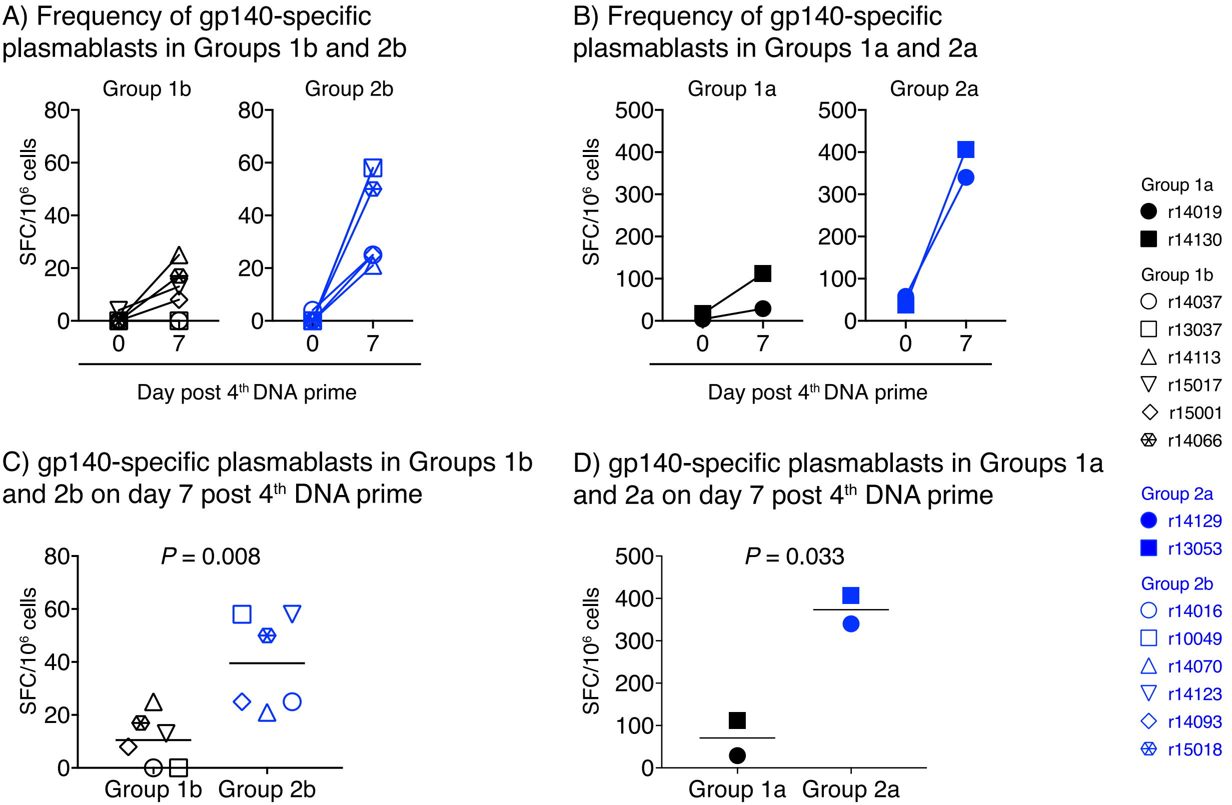

A) Frequency of gp140-specific plasmablasts in Groups 1b and 2b
Group 1b
Group 2b
SFC/10⁶ cells
0
20
40
60
80
0
7
Day post 4th DNA prime
B) Frequency of gp140-specific plasmablasts in Groups 1a and 2a
Group 1a
Group 2a
0
100
200
300
400
500
Day post 4th DNA prime
C) gp140-specific plasmablasts in Groups 1b and 2b on day 7 post 4th DNA prime
P = 0.008
Group 1b
Group 2b
D) gp140-specific plasmablasts in Groups 1a and 2a on day 7 post 4th DNA prime
P = 0.033
Group 1a
Group 2a
Group 1a
r14019
r14130
Group 1b
r14037
r13037
r14113
r15017
r15001
r14066
Group 2a
r14129
r13053
Group 2b
r14016
r10049
r14070
r14123
r14093
r15018
