# Supplementary material for: Vaccine protection against rectal acquisition of SIVmac239 in rhesus macaques
Source: PLoS Pathog. 2019 Sep 30;15(9):e1008015. doi: 10.1371/journal.ppat.1008015 (PMC6791558; doi:10.1371/journal.ppat.1008015)
Supplement: S9 Fig — A-B) Three vaccinees in Group 1b and two in Group 2b resisted 6 IR challenges with SIVmac239. PBMCs from these animals were obtained 2.3 weeks after the 6th SIV exposure and used in ICS assays containing peptides covering the entire SIVmac239 proteome. The total magnitude of vaccine-induced SIV-specific CD8+ (A) and CD4+ (B) T-cell responses measured at this timepoint was compared to that measured at the week of the 1st SIV challenge. The percentages of responding CD8+ T cells shown in the y-axes were calculated by adding the background-subtracted frequencies of positive responses producing any combination of IFN-γ, TNF-α, and CD107a. C) Two vaccinees in Group 1a and one in Group 2a resisted 6 IR challenges with SIVmac239. PBMCs from these animals were obtained 2.3 weeks after the 6th SIV exposure and stained with a fluorochrome-labeled Mamu-A*01/Gag181-189CM9 tetramer. The frequency of tetramer+ CD8+ T-cells measured at this time point was compared to that measured at the week of the 1st SIV challenge. Each symbol denotes one vaccinee. P-values were calculated using Welch’s t-test. (PDF) [file ppat.1008015.s009.pdf]

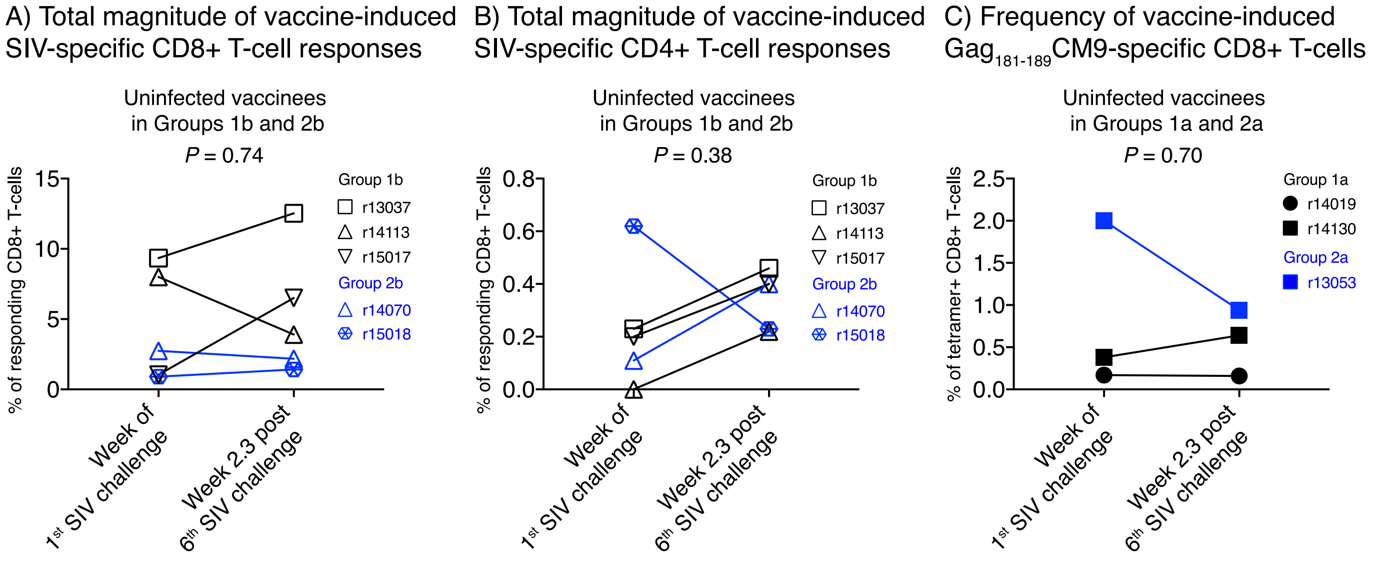

A) Total magnitude of vaccine-induced SIV-specific CD8+ T-cell responses
Uninfected vaccinees in Groups 1b and 2b
P = 0.74
% of responding CD8+ T-cells
15
10
5
0
Group 1b
r13037
r14113
r15017
Group 2b
r14070
r15018
Week of 1st SIV challenge
Week 2.3 post 6th SIV challenge
B) Total magnitude of vaccine-induced SIV-specific CD4+ T-cell responses
Uninfected vaccinees in Groups 1b and 2b
P = 0.38
% of responding CD8+ T-cells
0.8
0.6
0.4
0.2
0.0
Group 1b
r13037
r14113
r15017
Group 2b
r14070
r15018
Week of 1st SIV challenge
Week 2.3 post 6th SIV challenge
C) Frequency of vaccine-induced Gag181-189CM9-specific CD8+ T-cells
Uninfected vaccinees in Groups 1a and 2a
P = 0.70
% of tetramer+ CD8+ T-cells
2.5
2.0
1.5
1.0
0.5
0.0
Group 1a
r14019
r14130
Group 2a
r13053
Week of 1st SIV challenge
Week 2.3 post 6th SIV challenge
