# Supplementary material for: Vaccine protection against rectal acquisition of SIVmac239 in rhesus macaques
Source: PLoS Pathog. 2019 Sep 30;15(9):e1008015. doi: 10.1371/journal.ppat.1008015 (PMC6791558; doi:10.1371/journal.ppat.1008015)
Supplement: S10 Fig — (PDF) [file ppat.1008015.s010.pdf]

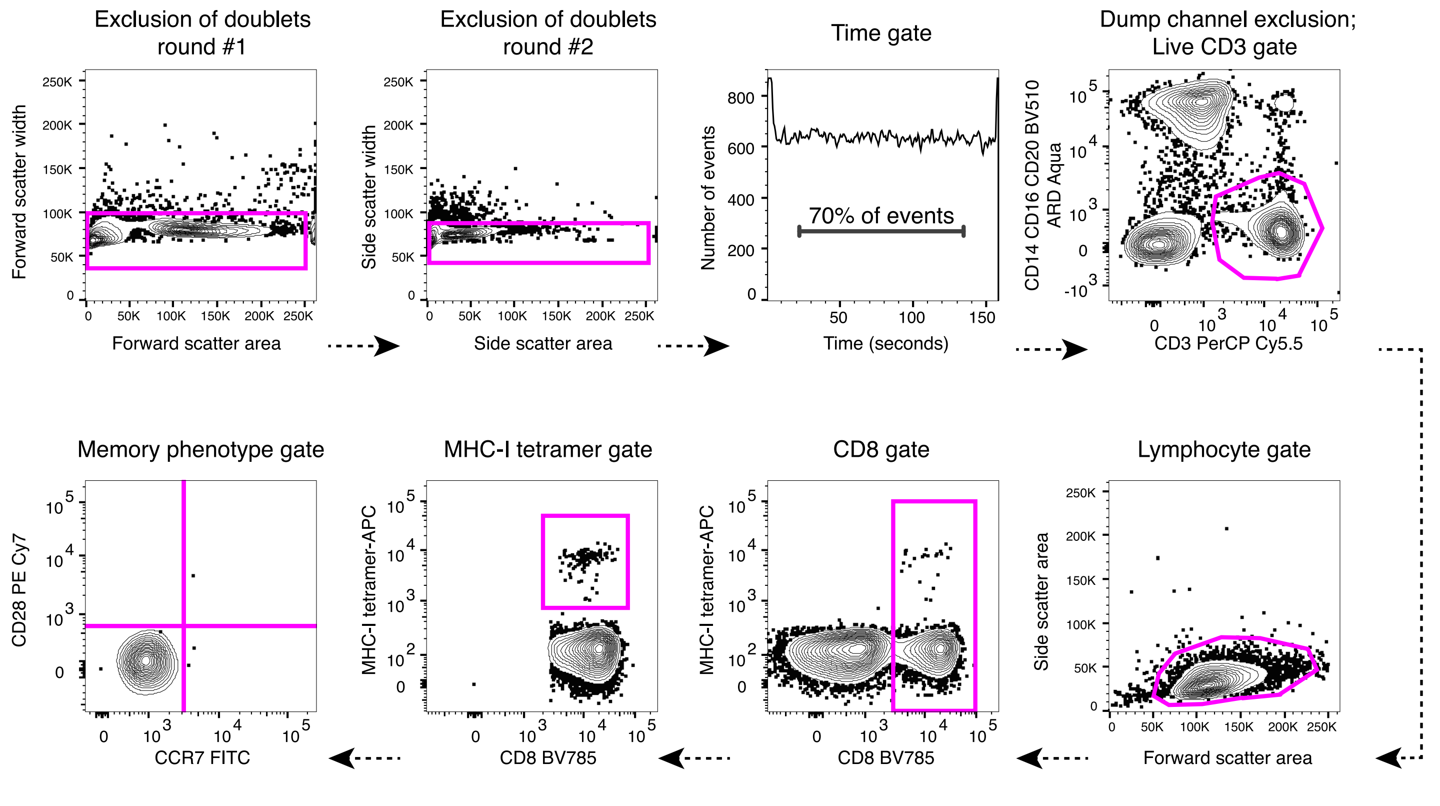

Exclusion of doublets round #1
Forward scatter width
Forward scatter area
Exclusion of doublets round #2
Side scatter width
Side scatter area
Time gate
Number of events
70% of events
Time (seconds)
Dump channel exclusion; Live CD3 gate
CD14 CD16 CD20 BV510 ARD Aqua
CD3 PerCP Cy5.5
Memory phenotype gate
CD28 PE Cy7
CCR7 FITC
MHC-I tetramer gate
MHC-I tetramer-APC
CD8 BV785
CD8 gate
MHC-I tetramer-APC
CD8 BV785
Lymphocyte gate
Side scatter area
Forward scatter area
