# Supplementary material for: Vaccine protection against rectal acquisition of SIVmac239 in rhesus macaques
Source: PLoS Pathog. 2019 Sep 30;15(9):e1008015. doi: 10.1371/journal.ppat.1008015 (PMC6791558; doi:10.1371/journal.ppat.1008015)
Supplement: S11 Fig — (PDF) [file ppat.1008015.s011.pdf]

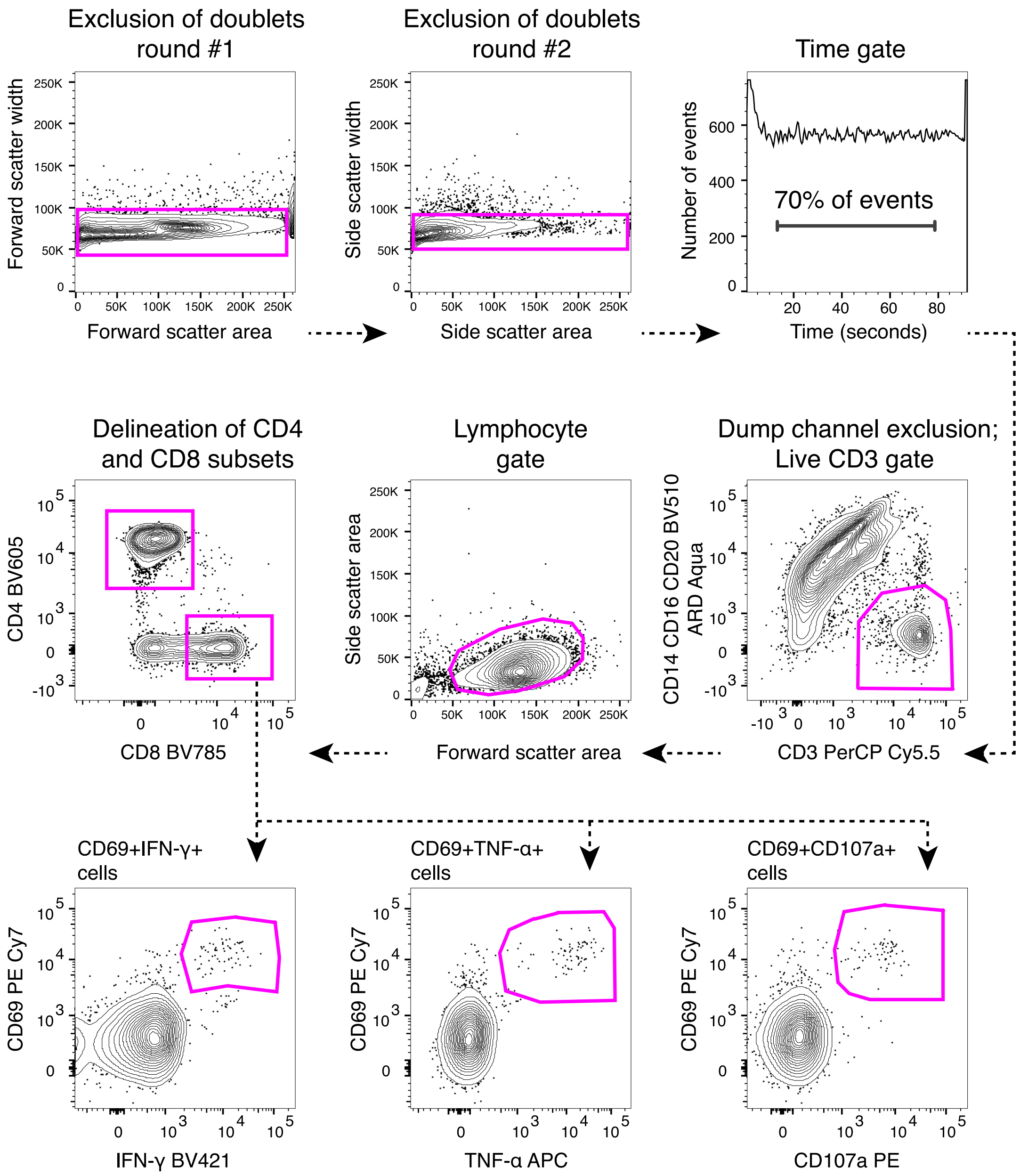

Exclusion of doublets round #1
Forward scatter width
Forward scatter area
250K
200K
150K
100K
50K
0
50K
100K
150K
200K
250K
Exclusion of doublets round #2
Side scatter width
Side scatter area
Time gate
Number of events
Time (seconds)
600
400
200
0
20
40
60
80
70% of events
Delineation of CD4 and CD8 subsets
CD4 BV605
CD8 BV785
Lymphocyte gate
Side scatter area
Forward scatter area
Dump channel exclusion; Live CD3 gate
CD14 CD16 CD20 BV510 ARD Aqua
CD3 PerCP Cy5.5
CD69+IFN-γ+ cells
CD69 PE Cy7
IFN-γ BV421
CD69+TNF-α+ cells
CD69 PE Cy7
TNF-α APC
CD69+CD107a+ cells
CD69 PE Cy7
CD107a PE
